# Single-Cell RNA-Sequencing Shift in the Interaction Pattern Between Glioma Stem Cells and Immune Cells During Tumorigenesis

**DOI:** 10.3389/fimmu.2020.581209

**Published:** 2020-10-08

**Authors:** You Zhai, Guanzhang Li, Renpeng Li, Yuanhao Chang, Yuemei Feng, Di Wang, Fan Wu, Wei Zhang

**Affiliations:** ^1^Department of Molecular Neuropathology, Beijing Neurosurgical Institute, Capital Medical University, Beijing, China; ^2^Department of Neurosurgery, Beijing Tiantan Hospital, Capital Medical University, Beijing, China; ^3^China National Clinical Research Center for Neurological Diseases, Beijing, China; ^4^Chinese Glioma Genome Atlas Network and Asian Glioma Genome Atlas Network, Beijing, China

**Keywords:** glioma stem cell, T cell, single cell sequence, immunosuppression, tumorigenesis

## Abstract

Glioblastoma is one of the most common neoplasms in the central nervous system characterized by limited immune response and unlimited expansion capability. Cancer stem cells (GSCs), a small fraction of the tumor cells, possess a pivotal regulation capability in the tumor microenvironment with a superior proliferation ability. We aimed to reveal the interaction between glioma stem cells (GSCs) and immune cells during tumorigenesis. Single-cell sequencing data from seven surgical specimens of glioblastoma patients and patient-derived GSCs cocultured with peripheral leukocytes were used for the analysis. Cell grouping and trajectory analysis were performed using Seurat and Monocle 3 packages in R software. The gene set of Cancer Genome Anatomy Project was used to define different cell types. Cells with the ability of proliferation and differentiation in glioblastoma tissue were defined as GSCs, which had a similar expression pattern to that in the GSCs *in vitro*. Astrocytes in glioblastoma were mainly derived from differentiated GSCs, while oligodendrocytes were most likely to be derived from different precursor cells. No remarkable evolutionary trajectory was observed among the subgroups of T cells in glioblastoma. The immune checkpoint interaction between GSCs and immune cells was changed from stimulatory to inhibitory during tumorigenesis. The patient-derived GSCs system is an ideal model for GSC research. The above research revealed that the interaction pattern between GSC glioma stem cells and immune cells during tumorigenesis provides a theoretical basis for GSC glioma stem cell-targeted immunotherapy.

## Introduction

Glioblastoma (GBM) is the most lethal type of intracranial malignancy (1). The median survival is about 14.4 months, and the overall survival varies from 3 months to 3 years (2). Among the many factors that contribute to poor outcomes, the existence of glioma stem cells (GSCs) and the immunological “cold tumor” status are considered to be two major pivotal ones (3, 4).

For the past few years, the dysfunction and poor infiltration of T cells in GBM tissue have become a major factor associated with poor prognosis according to a consensus (5). Several strategies for T cell dysfunction in GBM tissue have been described (6). Although T cells are overwhelmed by tumor cells in GBM, not all tumor cells possess the ability of immune regulation. Thus, studying the interaction between tumor cells and T cells may be a new direction in tumorigenesis research.

In recent years, GSCs have become a novel hot spot due to their tumorigenesis and immune regulation capabilities (7). GSCs play a pioneering immunosuppressive role at the time of tumor initiation and gradually lose these capabilities during differentiation to astrocytes and oligodendrocytes. Further, GSCs are considered to be extremely resistant to therapy (8), leading to the failure of multiple treatments, including immunotherapy. Therefore, revealing the interaction between GSCs and T cells may provide novel immunotherapeutic strategies for glioma.

In this article, peripheral T cells and GSC coculture models were built *in vitro* to simulate the initial state of tumor. Taking advantage of the single-cell sequencing data, we were able to identify different subtypes of cells and further analyze the evolutionary relationship between each subtype of tumor cells, as well as immune cells. First, we identified subtypes of GSCs in surgical specimens according to the high proliferation characteristics. Then, we constructed the coculture model of T cells and GSCs. We cross-validated the DNA expression patterns in the GCSs in the established coculture model and surgical specimens. An ideal similarity was detected. Further, we depicted an evolution routine for GSCs in surgical specimens. The astrocytes showed a strong evolutionary relation with GSCs. Since T cells showed various characteristics in those two data sources, we defined the coculture model as the initial stage of tumor progression and the specimens as the advanced stage of tumor. Finally, we simulated the fold change of the immune checkpoint in both T cells and GSCs in those two data sources. The inhibiting checkpoint resulted in an advanced tumor stage. Above all, the *in vitro* model is an ideal tool for unveiling the interaction between peripheral T cells and GSCs, simulating the early microenvironment during tumorigenesis.

## Materials and Methods

### Isolation and Culture of Primary Cells

Tumor tissues obtained during surgery were immediately immersed in the medium and transported to the laboratory on ice for further processing. The tissue was cleaned and shredded mechanically. The tissue was then enzymatically digested into single cells using trypsin. The single cells were filtered using a 200-mesh filter and centrifuged (400 g) for 5 min. After treating the cells with red blood cell lysis, they were centrifuged again. The obtained cells were cultured in a serum-free medium containing DMEM/F12 (Gibco) supplemented with B27 (Gibco), basic fibroblast growth factor (bFGF, 20 ng/mL), epidermal growth factor (EGF, 20 ng/mL), and heparin (2.5 mg/mL). Growth factors (bFGF and EGF) were added twice a week. Primary GSCs were enzymatically dissociated into single cells using Accutase (Sigma Aldrich) and thereafter routinely cultured in the serum-free medium that was replaced every 4–6 days. The stemness of GSCs was verified by multidirectional differentiation immunofluorescence staining (**Figure 2A**).

Normal peripheral blood lymphocytes were obtained from healthy adult male donors. Isolation of peripheral blood T cells was performed following the protocol as previously described (9). In brief, peripheral blood mononuclear cells (PBMCs) were separated by density gradient centrifugation with Lymphoprep (STEMCELL). The PBMCs were resuspended in EasySep™ Buffer (STEMCELL), and T cells were isolated following the manufacturer's instruction (EasySep™ Human T Cell Isolation Kit, STEMCELL). T cells were identified by CD3 staining flow cytometry (Figure 2A).

Peripheral blood T cells were cocultured with GSCs for 24 h the day after isolation without CD3/CD28 stimulation. 2 × 10^6^ T cells, together with 1 × 10^6^ GSCs, were directly mixed and resuspended in ImmunoCult™-XF T Cell Expansion Medium (STEMCELL) and were cocultured in a 37°C 5% CO_2_ incubator.

### Construction of a Single-Cell RNA-Sequencing Library

Single-cell RNA sequencing library construction of the tissue specimens obtained from GBM patients has been described in detail in our previous research (10). The cell preparation for coculture cellular model was done strictly in accordance with the official documentation of 10 × Genomics (https://support.10xgenomics.com). Single-cell RNA sequencing was performed using Illumina (HiSeq 2000) according to the manufacturer's instructions by Novogene (Beijing, China).

### Cell Clustering Using Seurat

The cell clustering in GBM patients and coculture model of primary normal peripheral blood lymphocytes and GSCs was performed by the R package Seurat (version 3.0, https://satijalab.org/seurat/). Batch effect was removed before the clustering in GBM patients. Subsequently, the cell clustering process in GBM patients and the coculture model were done in the same way. Firstly, cells that have had unique feature counts over 7,500 or <200 and >15% mitochondrial count were removed. Subsequently, after normalizing the data, non-linear dimensional reduction of cells was carried out using UMAP with the default parameters. Finally, the cluster biomarkers were also obtained. In addition, the t-SNE method was also used to verify the reliability of cell grouping of the UMAP method (Supplementary Figure 7).

### Identification of Cell Clusters

The Cancer Genome Anatomy Project Serial Analysis of Gene Expression (CGAP_SAGE_QUARTILE) was launched to determine the genetic fingerprints of normal, premalignant, and malignant tumor cells based on the transcriptome characteristics of cells (PMID: 10933042). Identification of cell clusters was performed using CGAP_SAGE_QUARTILE analysis in DAVID portal (https://david.ncifcrf.gov/) according to the cluster biomarkers.

### Functional Enrichment Analysis of Cell Clusters

Gene Ontology (GO) enrichment and KEGG pathway analysis of cell clusters were used to identify the biological significance of each cell type. GO and KEGG pathway analyses were conducted using the cluster biomarkers.

### Single-Cell Trajectory Analysis

The R package Monocle 3 was applied to order cells in pseudotime along a trajectory (https://cole-trapnell-lab.github.io/monocle3). After clustering the cells using the above method, the dimensionality was reduced and the results were visualized using the UMAP method. Subsequently, the cells were ordered according to their progress through the developmental program. Monocle measures this progress in pseudotime. In this study, single-cell trajectory analysis of cell subtypes was performed as needed.

### Software Availability

Statistical analyses and drawing were performed using the R program (https://www.r-project.org/, version: 4.0), TBtools software (version: 0.67), Java software (version: 12.0.1), and Microsoft office 2016. The Sankey diagram was drawn using online tools (http://sankeymatic.com/build/).

## Results

### Identification of Glioma Stem Cells in GBM Tissue Samples

**Figure 1:**
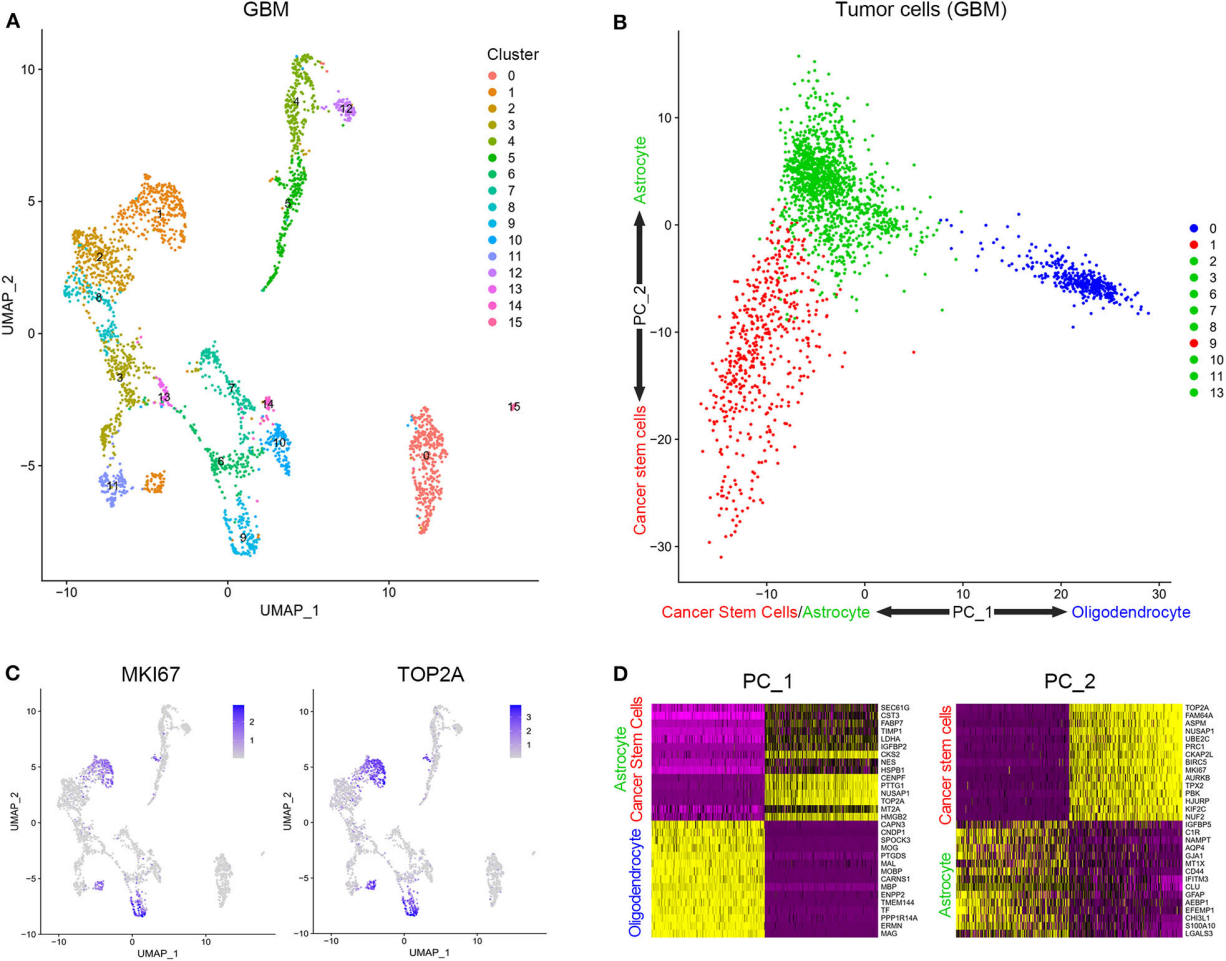
Stratification and identification of cells from surgical specimens. **(A)** The subgroups of cells in surgical specimens. **(B)** The expression level of proliferation-associated biomarkers in different cell subgroups. **(C)** The PCA analysis of tumor cells in surgical samples. **(D)** Genes related to PC1 and PC2.

Cells from tissue samples of 7 GBM patients were grouped into 16 clusters according to a single-cell sequencing data (Figure 1A). Based on CGAP_SAGE_QUARTILE, cell types of 16 clusters were identified according to their gene expression pattern. Clusters 1 and 9 were identified as GSCs. Clusters 4, 5, 12, 14, and 15 were identified as immune cells (Supplementary Figure 1). Clusters 0, 2, 3, 6, 7, 8, 10, 11, and 13 were identified as tumor cells (Supplementary Figure 1). To further identify GSCs from these 16 clusters, we examined the proliferation of cells. As a result, clusters 1 and 9 possessed the significantly increased expression of proliferation markers KI67 and TOP2A (Figure 1B). In addition, clusters 1 and 9, together with the rest subgroups of tumor cells (Clusters 0, 2, 3, 6, 7, 8, 10, 11, and 13), were further engaged in principal component analysis (PCA). Cells in clusters 1 and 9 possessed low PC-1 and PC-2 values while cells in clusters 2, 3, 6, 7, 8, 10, 11, and 13 possessed low PC-1 and high PC-2 values. On the contrary, cluster 0 possessed high PC-1 and high PC-2 values (Figure 1C). The genes that were positively correlated with PC-1 values were mainly oligodendrocyte markers, while those that were negatively correlated with PC-1 values were mainly cancer stem cells and astrocyte markers. On the other hand, the genes that were positively correlated with PC-2 values were mainly astrocyte markers and those that were negatively correlated with PC-2 values were mainly cancer stem cells markers (Figure 1D and Supplementary Table 1). In short, clusters 1 and 9 containing a group of cells with high proliferation and differentiation abilities had the characteristics of cancer stem cells.

### The Coculture Model Was Built to Simulate the Initial State of Tumor Development

**Figure 2:**
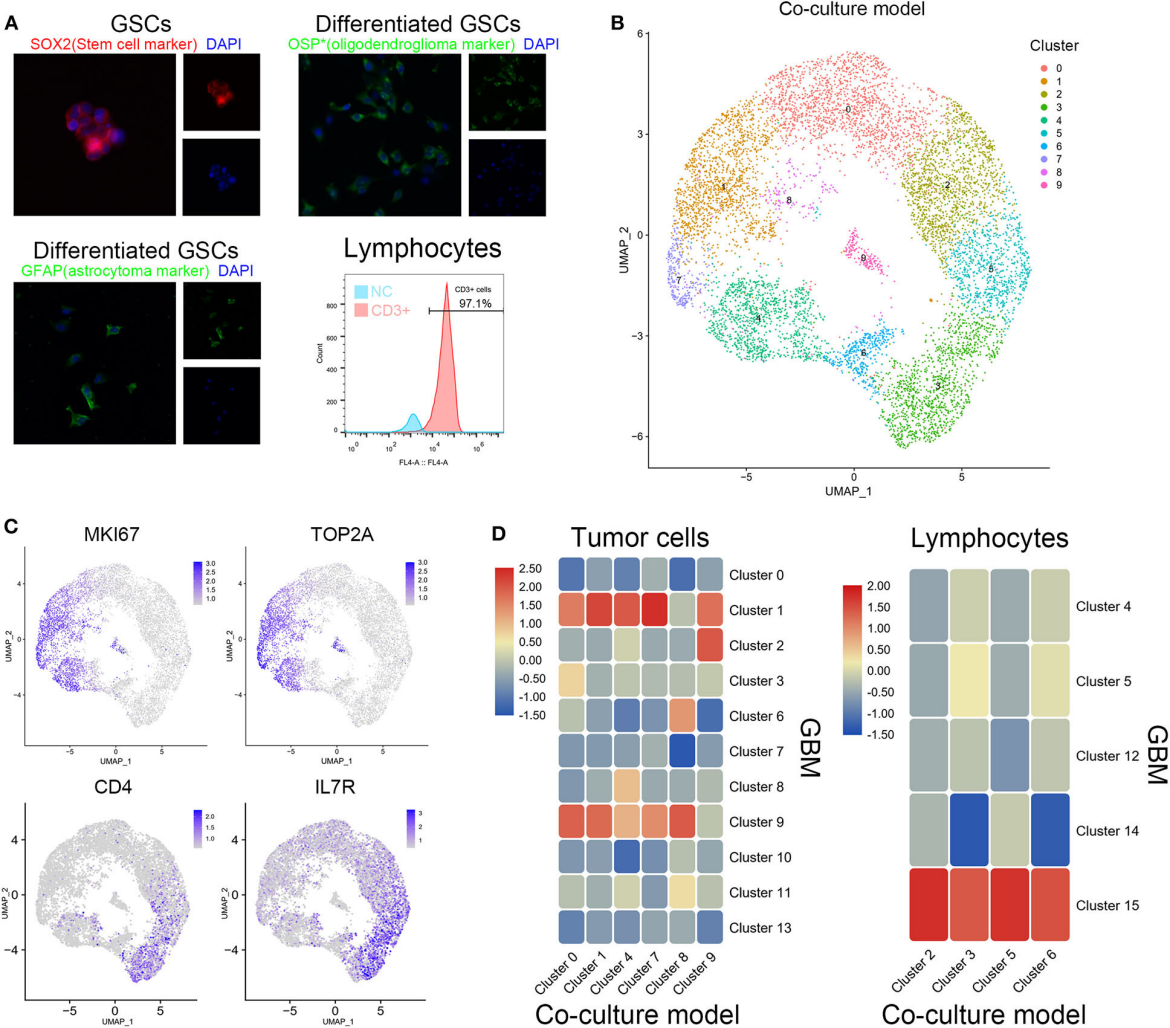
Similarity of cell grouping in the coculture model and surgical specimens. **(A)** The GSCs and T cells were verified by immunofluorescence staining and flow cytometry. OSP: oligodendrocyte specific protein. **(B)** The subgroups of cells in the coculture model. **(C)** Markers of proliferation and immunology in different cell groups. **(D)** The similarity of glioma stem cells and lymphocytes in the coculture model and surgical specimens.

Patient-derived GSCs and peripheral blood lymphocytes from healthy adults were cocultured to simulate the initial state of tumor. After identification by cell surface markers, patient-derived GSCs and peripheral blood lymphocytes were mixed (1:2) and cocultured (Figure 2A). Single-cell sequencing of the mixed cells was performed after 12 h of coculture. The 10 clusters of the cocultured cells are indicated in Figure 2B. As expected, cell clusters could be divided into GSCs and lymphocytes according to their proliferation rate and the expression of immune cell markers. Clusters 0, 1, 4, 7, 8, and 9 with high expression of KI67 and TOP2A were identified as GSCs. Clusters 2, 3, 5, and 6 were considered to be T cells with their extracellular markers, CD4 and IL7R (Figure 2C). We further compared the similarity between GSCs and T cells from coculture model and GBM samples. As shown in Figure 2C, clusters 0, 1, 4, 7, 8, and 9 in the coculture model possessed the similar gene expression characteristics with clusters 1 and 9 in GBM samples. Clusters 2, 3, 5, and 6 in the coculture model were similar to cluster 15 in GBM samples (Figure 2D). The list of cell markers of all clusters is uploaded in Supplementary Table 2. Meanwhile, cell types of cocultured cells were also identified based on CGAP_SAGE_QUARTILE. The cell types identified using the CGAP_SAGE_QUARTILE were highly consistent with those defined using the cell markers (Supplementary Figure 2).

### Stem Cells in the Coculture Model and GBM Samples Showed Highly Similar Expression Characteristics

**Figure 3:**
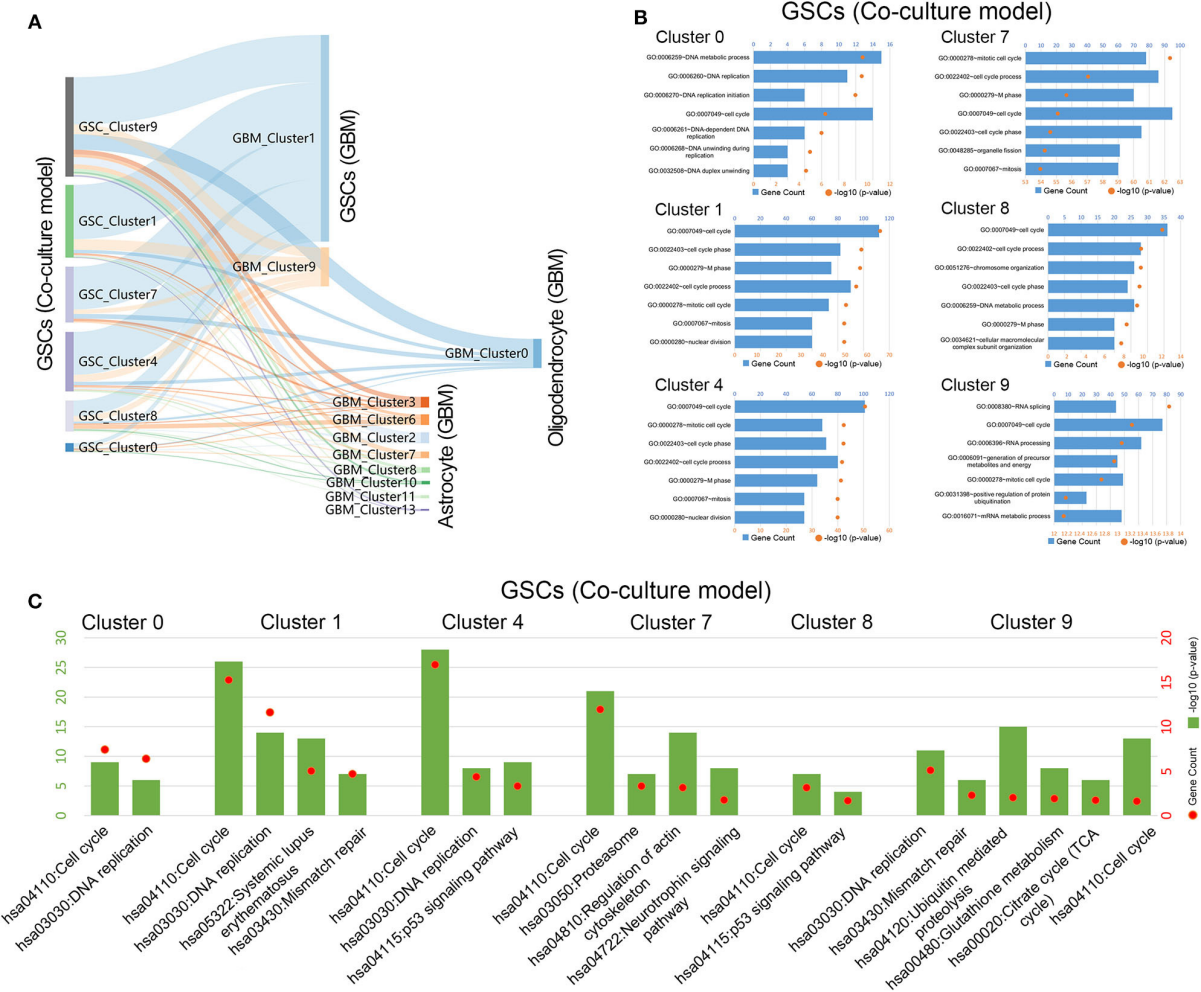
Gene function analysis of glioma stem cells in the coculture model. **(A)** Gene numbers simultaneously expressed in glioma stem cells in both the coculture model and surgical specimens. **(B)** Enrichment analysis of biological functions for glioma stem cells in the coculture model. **(C)** Path enrichment analysis for glioma stem cells in the coculture model.

To further explore the relationship between cells in coculture model and GBM samples, the correlation of the expression characteristics of tumor cells in these two groups was compared. As shown in Figure 3A, all clusters of GSCs in the coculture model possessed the majority of the coexpressed genes with clusters 1 and 9 in tumor specimens. Surely, GSCs in the coculture model also had some coexpressed genes with astrocytes as well as oligodendrocytes. Subsequently, functional enrichment analysis of tumor cells in the coculture model and GBM samples was performed using GO analysis and KEGG analysis. Stem cells in both groups of cells were characterized by high proliferation capacity (Figures 3B,C, Supplementary Figures 3, 4), while other non-stem tumor cells showed significantly different biological characteristics (Supplementary Figures 3–5). These results suggested that the patient-derived stem cells and the defined GSCs in GBM samples shared a high level of proliferation-related markers, as well as active proliferation pathways, indicating the ultimate proliferation capacity of these cells.

### Evolution Routine Can Be Described Between Glioma Stem Cells and Astrocytes in GBM Samples

**Figure 4:**
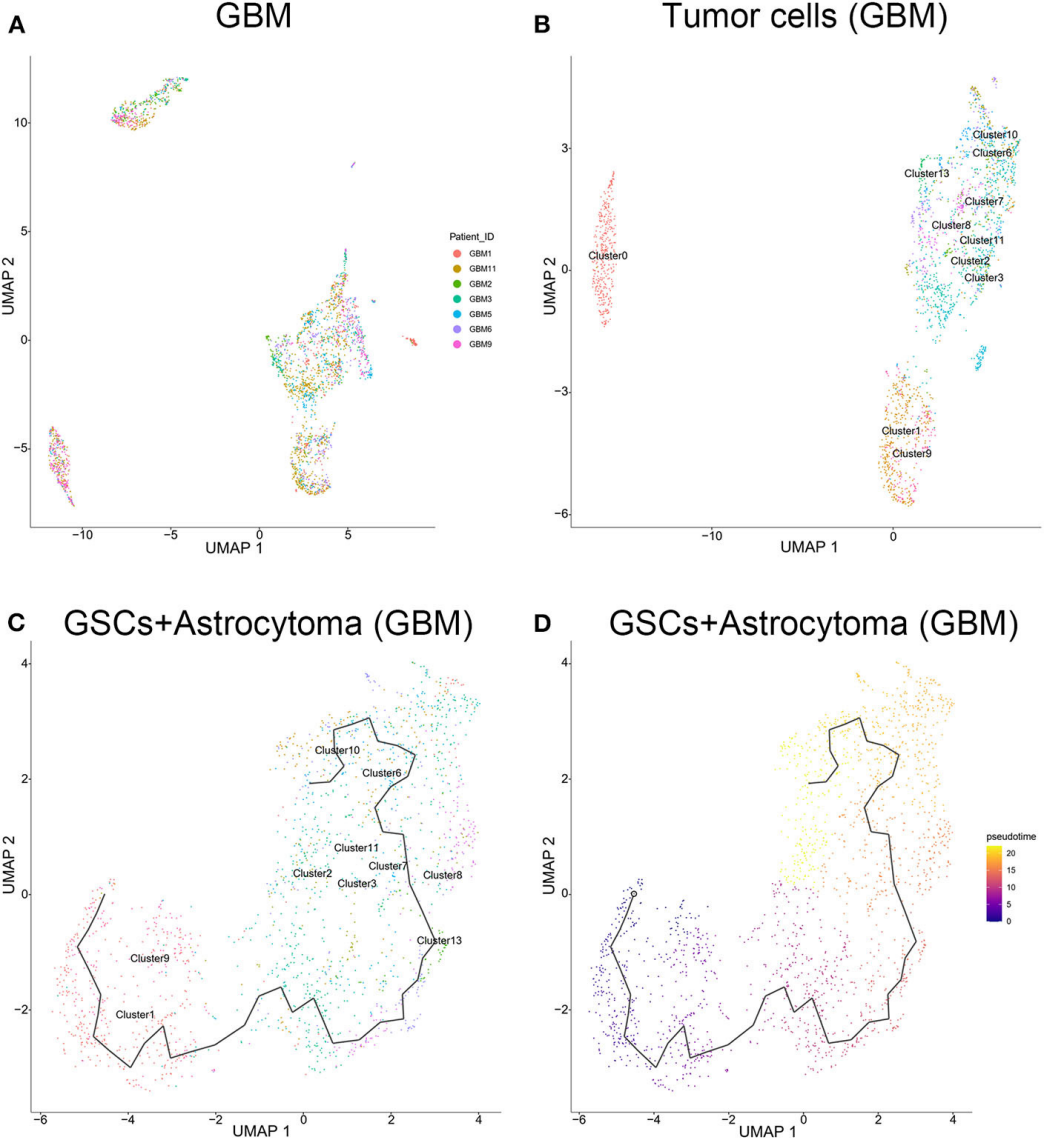
Evolution routine of tumor cells in surgical specimens. **(A)** No significant batch difference in tumor cells from different surgical specimens. **(B)** Tumor cell subgrouping in surgical specimens. **(C)** The evolution direction of tumor cells in surgical specimens. **(D)** The pseudo-time sequence of evolution of tumor cells in surgical specimens.

The tumor cells from the GBM samples were extracted for further study. The results showed that no significant batch effect of tumor cells has been observed among patients (Figure 4A). As mentioned above, clusters 0, 1, 2, 3, 6, 7, 8, 9, 10, 11, and 13 of GBM samples were identified as tumor cells, including GSCs (clusters 1 and 9) and astrocytes (clusters 2, 3, 6, 7, 8, 10, 11, and 13) and oligodendrocytes (cluster 0). As shown in Figure 4B, among the three groups of cells, the oligodendrocytes were relatively insular compared with the other two cell types. To further unveil the differentiation process from GSC to astrocytes or oligodendrocytes, trajectories of GBM tumor cells were calculated. The results showed GSC's evolution into astrocytes through a certain path in terms of evolution time (Figures 4C,D). However, as the subgroups of GSC and oligodendrocytes were far apart on the evolutionary route and there was no fundamental connection between those two cell types, we could conclude that there was no evolutionary relation between GSCs and oligodendrocytes (Supplementary Figure 6).

### T Cells Showed Differences in Biological Functions and Pathway Activation Between the Coculture Model and GBM Samples

**Figure 5:**
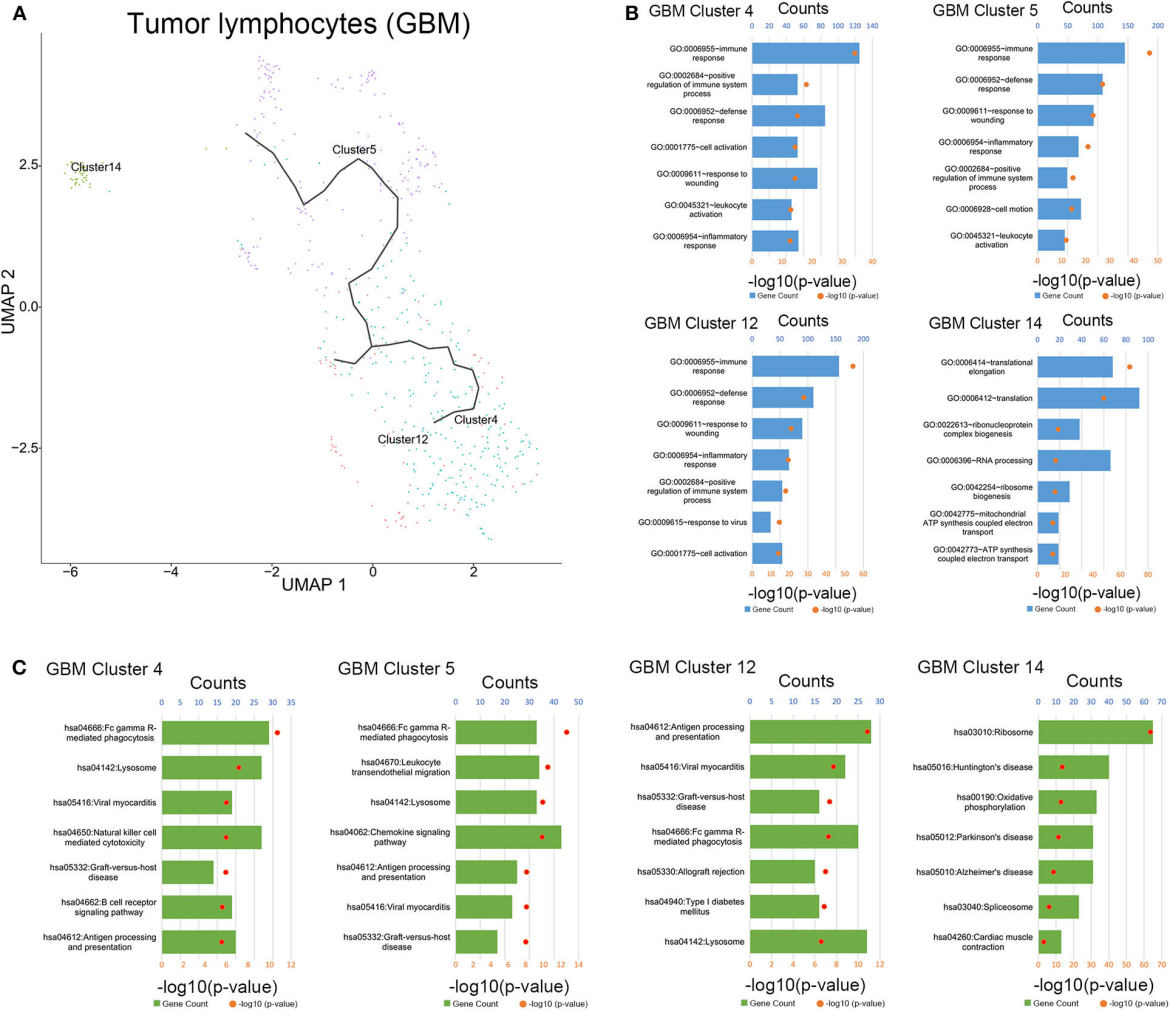
Evolution routine and function enrichment of lymphocytes in surgical specimens. **(A)** The evolution direction of lymphocytes in surgical specimens. **(B)** Enrichment analysis of biological functions for lymphocytes in the surgical specimens. **(C)** Pathway enrichment analysis for lymphocytes in the surgical specimens.

To clarify the evolutionary trajectory of T cells in tumors, trajectories of T cells in GBM were calculated. A clear evolutionary route could be found in T cells (Figure 5A). However, the cluster of initial T cells (Cluster 14) was not in the evolution path. The direction of evolution was difficult to determine. Subsequently, GO analysis and KEGG analysis were performed to reveal the biological functions of T cells in the coculture model and GBM samples (Figures 5B,C, Supplementary Figures 3–5). Different groups of T cells showed different biological functions and pathway activations.

### Immune Checkpoint Interaction Pattern Changed Significantly Between the Coculture Model and GBM Samples

**Figure 6:**
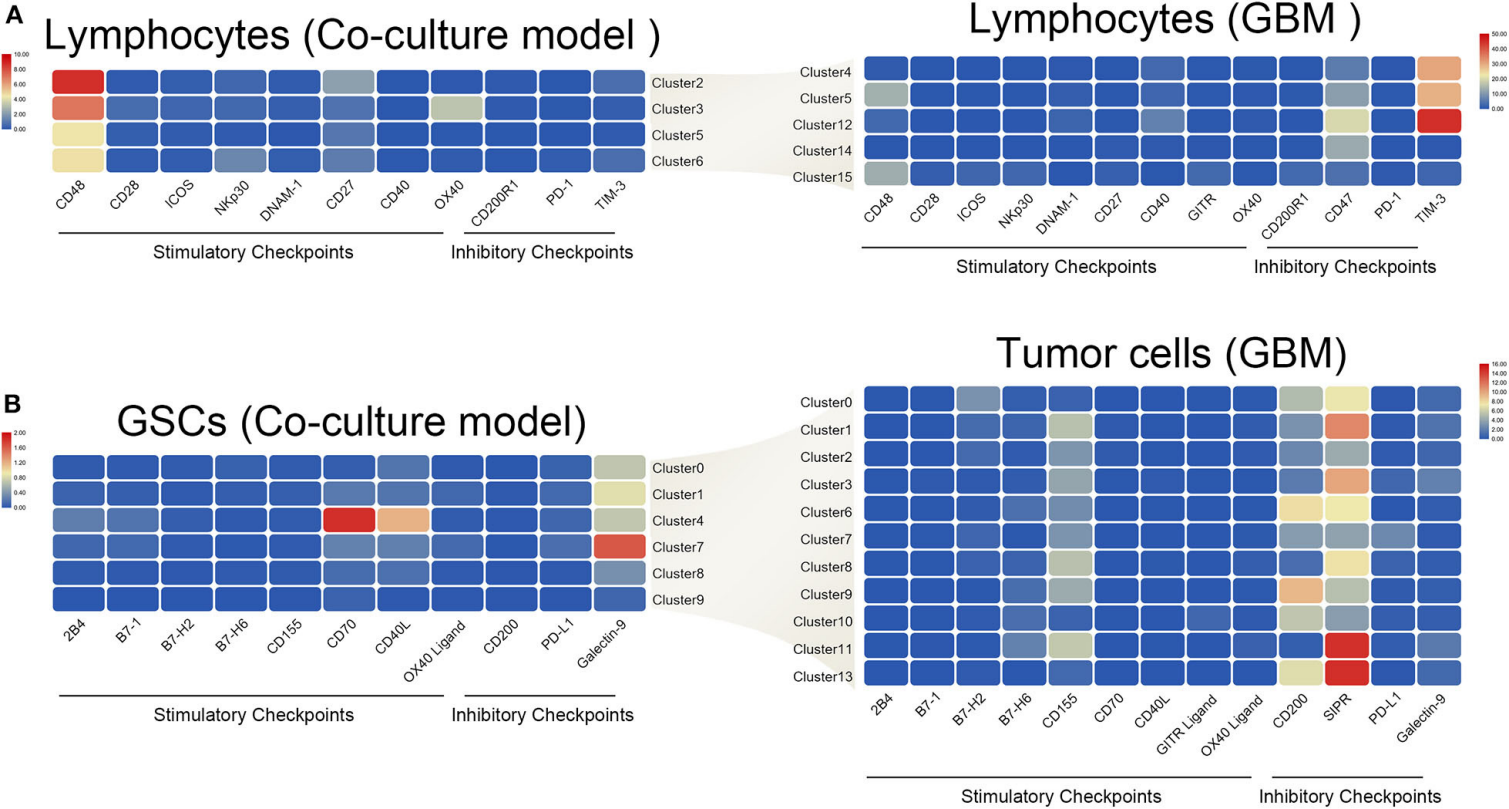
Differences in the interaction pattern between tumor cells and immune cells in early and late stages of glioma. Changes in expression of immune checkpoints and the corresponding receptors in both immune cells **(A)** and glioma stem cells **(B)**, in the early and late stages of glioma.

The interaction of immune checkpoint of T cells and tumor cells were analyzed in the coculture model and GBM samples separately. The stimulatory immune checkpoint genes were expressed mainly in T cells in the coculture model, while inhibitory immune checkpoint genes were enriched in T cells in GBM samples (Figure 6A). Similarly, tumor cells mainly expressed ligands of stimulatory immune checkpoints in the coculture model, while tumor cells in GBM samples mainly expressed ligands of inhibitory immune checkpoints (Figure 6B). Significant changes in this interaction model may reveal the causes of tumor immunosuppression in the microenvironmental status during tumorigenesis.

## Discussion

Numerous studies had confirmed the high level of immunosuppression during GBM processing, which contributes to the dysfunction of the infiltrated immune cells and immunotherapeutic failure (11). Recently, several studies underlined the importance of GSCs for the initiation of immune suppression during tumorigenesis (12, 13). However, the identification of cancer stem cells is challenging. The limited methods for cancer stem cell separation hindered research progress, although beads or flow separation, as well as the introduction of a special culture medium, has been widely used for cancer stem cell sorting. Single-cell sequencing has allowed us to perform multiple analysis of different cell types in a large number of specimens and in *in vitro* culture samples (14).

Thus, by means of single-cell sequencing using the GBM samples, cells with high proliferation and differentiative capacity were defined as GSCs. Similarly, single-cell sequencing data of the established coculture model of the patient-derived GSCs and human peripheral blood T cells were also analyzed. Comparative analysis showed high similarity between GSCs in GBM samples and those in the coculture model. In addition, we have also verified the similarity between these two populations based on the markers, biological function enrichment, and other parameters. It suggests that the coculture model we constructed can simulate the initial status of stem cells in tumors, which could be used in further research. Surprisingly, we found that there were few immune cells (cluster 15) in the surgical specimens that were highly similar to the peripheral blood lymphocytes in the coculture system (cluster 15). We speculated that it was due to the small amount of peripheral blood “contamination” caused by the operation. Since those lymphocytes may not be the original immune cells in the tumor, this phenomenon should be noted when identifying immune cell clusters using single-cell sequencing in the future.

The application of trajectory analysis using single-cell sequencing data in the evolution research has attracted more and more attention. Such technique has been applied to the evolution research of many tumors, e.g., liver cancer (15). Therefore, we used Monocle 3, the most commonly used tool for studying tumor evolution, to analyze the evolution of tumor cells in GBM samples. Our study revealed that GSCs had a differentiation ability. On the other hand, whether astrocytes and oligodendrocytes in tumors were directly originating from GSCs remains controversial. We unveiled that the astrocytes in the tumor were likely derived from GSC. On the contrary, oligodendrocytes showed significantly different characteristics from astrocytes. In addition to possessing significantly different gene expression characteristics, oligodendrocytes were less heterogeneous than astrocytes. Further, oligodendrocytes and astrocytes were proved to have different origins instead of both cell types originating from GSC. Unfortunately, our study did not find precursor cells of the oligodendrocytes. These research results provide a theoretical basis for the follow-up research and targeted therapy of cancer stem cells.

The immune modulating abilities of GSCs were attributed to inducing cytotoxic T cell (CTL) anergy/apoptosis and expansion of regulatory T cells (Treg) (16). Meanwhile, Tregs were well-known immune suppression cells (17). Nevertheless, an ideal model for simulation of the initial interactions between T cells and GSCs has not yet been reported. Thus, we suppose that the GSC and T cell coculture system might be an ideal model for simulating the early stages of tumorigenesis. Our analysis unveiled that the immune cells in GBM samples had a clear evolutionary trajectory. Clusters 4 and 5 were identified as tumor-associated macrophages. The evolution between clusters 4 and 5 may be the result of the transformation of M1 and M2. This suggests that the evolution of immune cells in the tumor microenvironment may play a role in tumor progression, although the particular mechanism remains unclear. Therefore, we defined the coculture model of GSCs and peripheral blood T cells as the early state of the tumor and the surgical samples of GBM patients as the advanced state of the tumor. By comparing the expression of immune checkpoint-related genes between these two stages, we found that both T cells and tumor cells had a preferential expression of the stimulatory immune checkpoints. However, in the advanced stages of tumors, these two types of cells expressed more suppressive immune checkpoints, which finally evolved into the state of the immune microenvironment consistent with the consensus. A further in-depth study of this transition process may provide new treatment ideas for immunotherapy of gliomas.

In summary, our research confirmed the existence of a group of cells possessing highly proliferative and differentiative capability in the tumor, which are called glioma stem cells. In addition, it also established a reliable *in vitro* model for glioma stem cell research. Our research revealed the evolution of glioma stem cells and the changes in immune status, which can provide new ideas for immunotherapy of gliomas.

## Data Availability Statement

The datasets for this study can be found in the CGGA portal (http://www.cgga.org.cn/).

## Ethics Statement

Sample collection and data analyses were approved by Beijing Tiantan Hospital institutional review board (IRB). The patients/participants provided their written informed consent to participate in this study.

## Author Contributions

WZ: conception, supervision, and design of this article. YZ and GL: data analysis and editing the manuscript. RL, YC, and YF: data collection and organization of Single-Cell RNA-Sequencing data. YZ, GL, DW, and FW: isolation and culture of primary cells. All authors contributed to the article and approved the submitted version.

## Conflict of Interest

The authors declare that the research was conducted in the absence of any commercial or financial relationships that could be construed as a potential conflict of interest.
