# Supplementary material for: Single-Cell RNA-Sequencing Shift in the Interaction Pattern Between Glioma Stem Cells and Immune Cells During Tumorigenesis
Source: Front Immunol. 2020 Oct 8;11:581209. doi: 10.3389/fimmu.2020.581209 (PMC7580180; doi:10.3389/fimmu.2020.581209)
Supplement: Supplementary file 1 [file Table_1.DOCX]

Supplementary Material

## Supplementary Figures


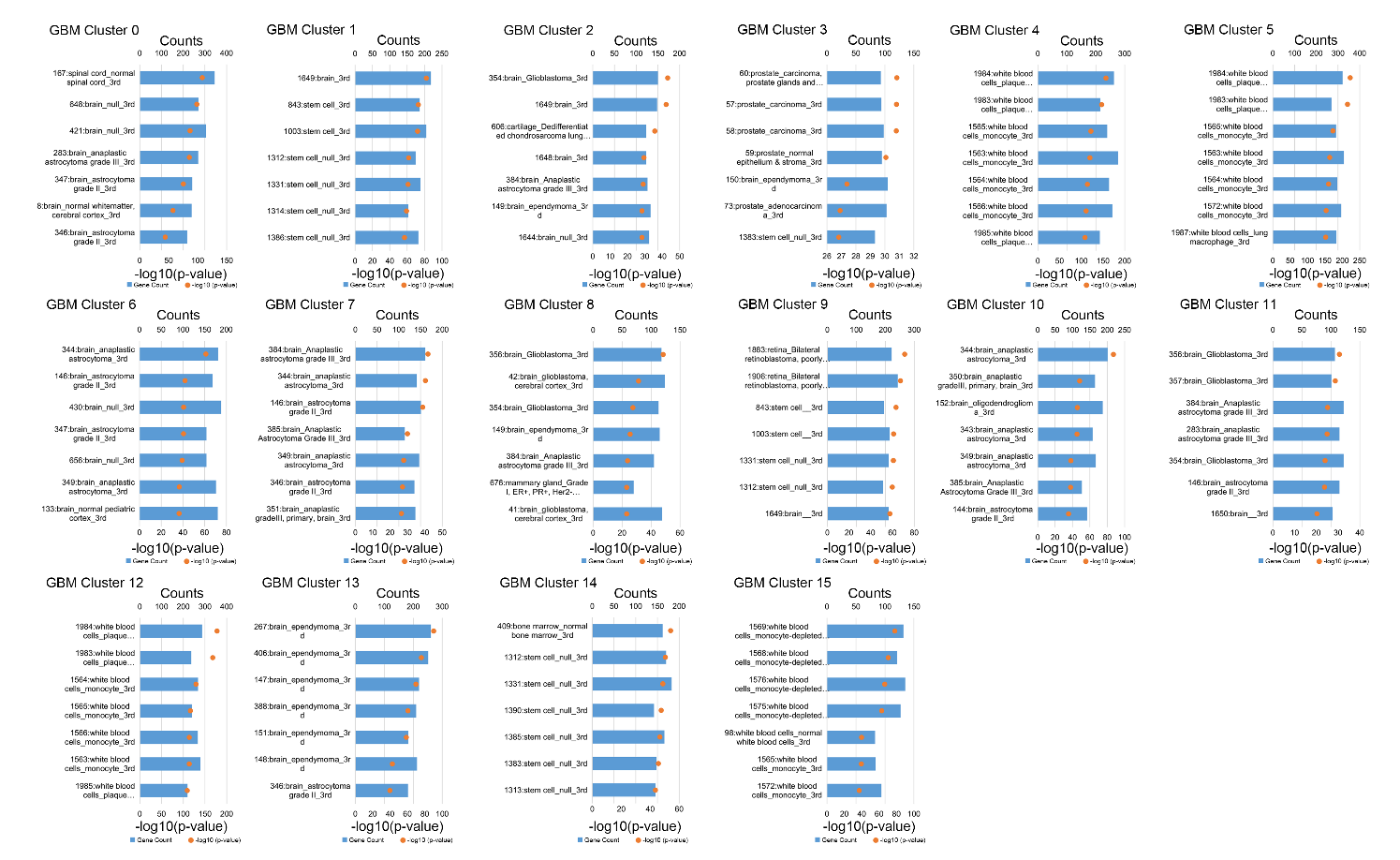


**Supplementary Figure 1.** Definition of each cell sub-groups in surgical specimens, based on CGAP_SAGE_QUARTILE


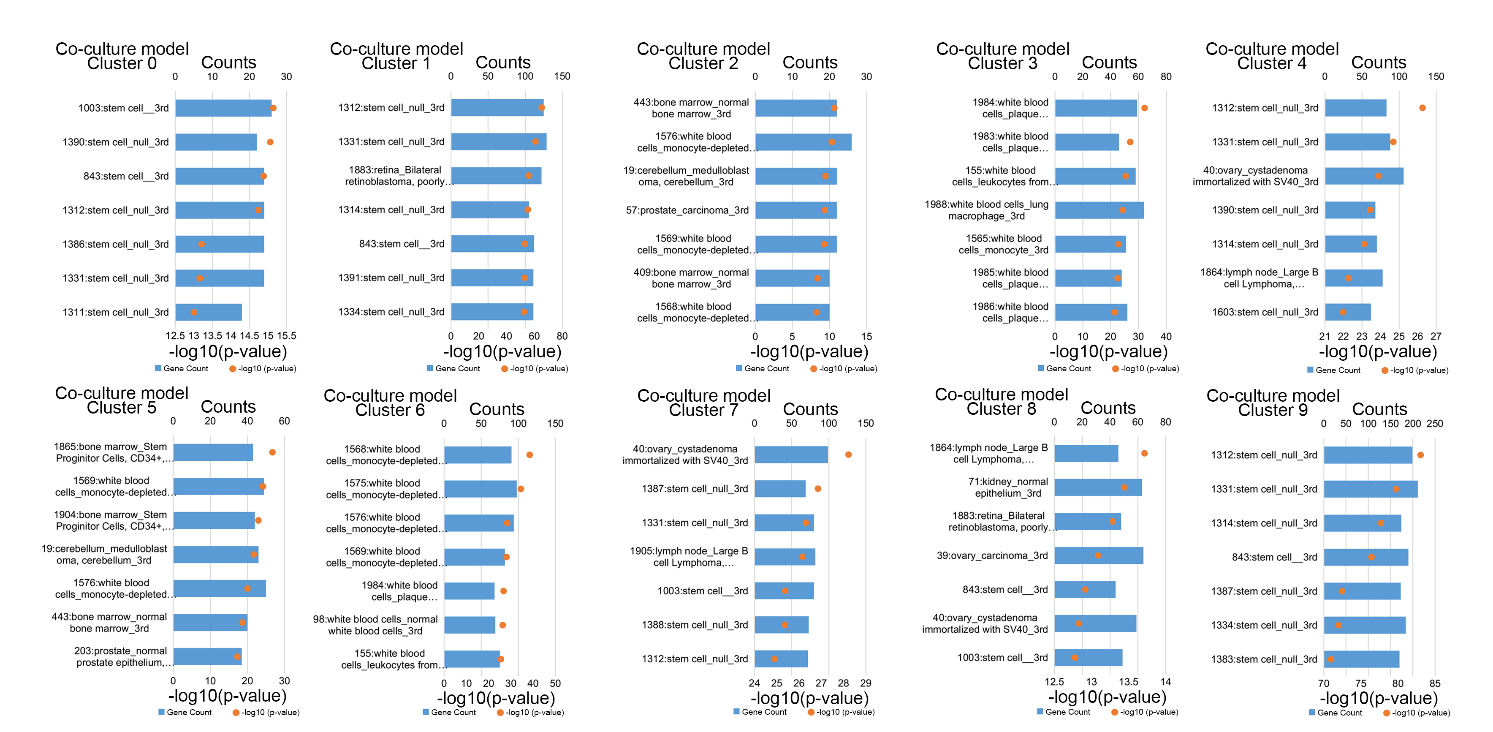


**Supplementary Figure 2.** Definition of each cell sub-groups in the co-culture model, based on CGAP_SAGE_QUARTILE


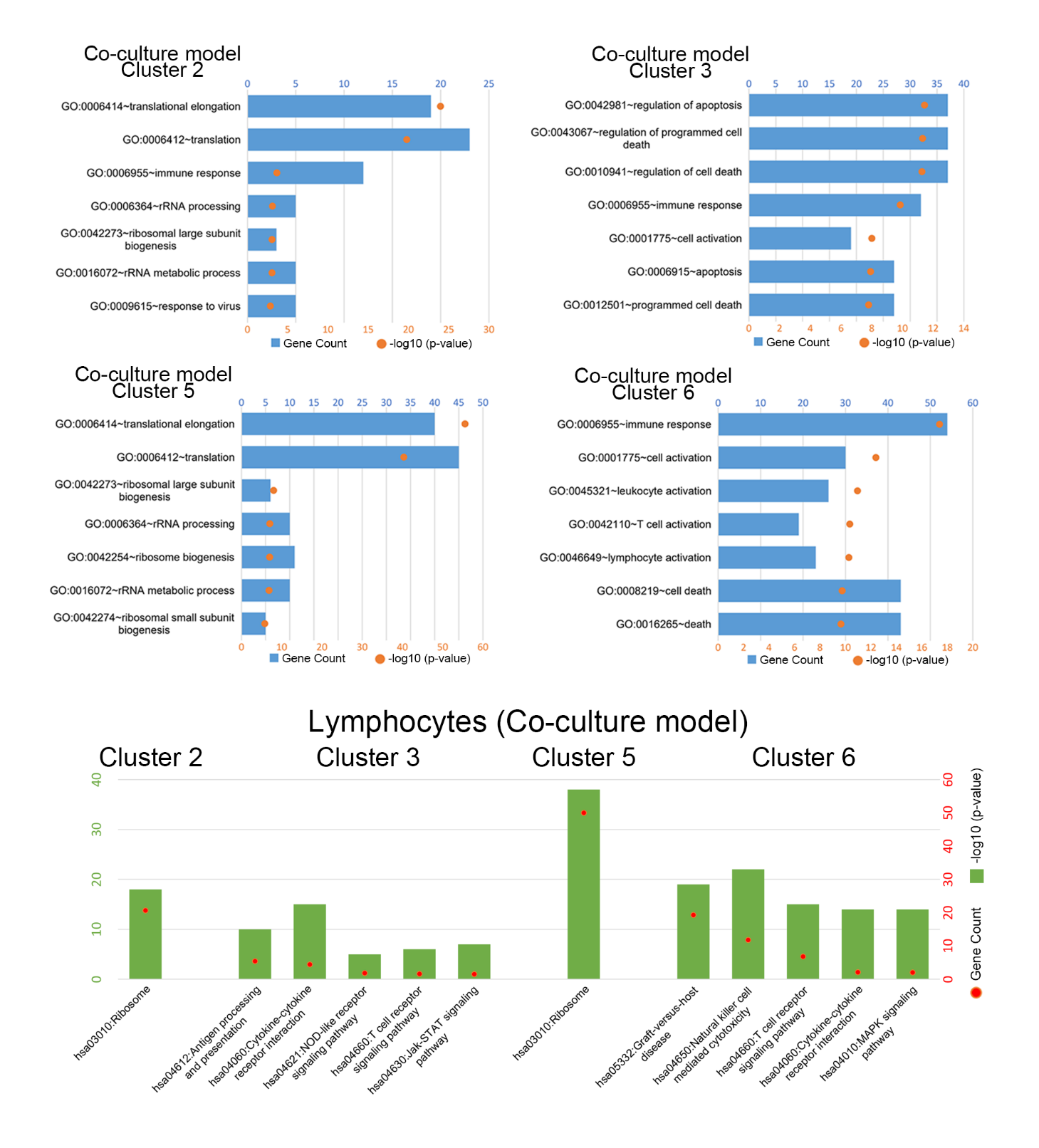


**Supplementary Figure 3.** Definition of each cell sub-groups in the co-culture model, based on CGAP_SAGE_QUARTILE


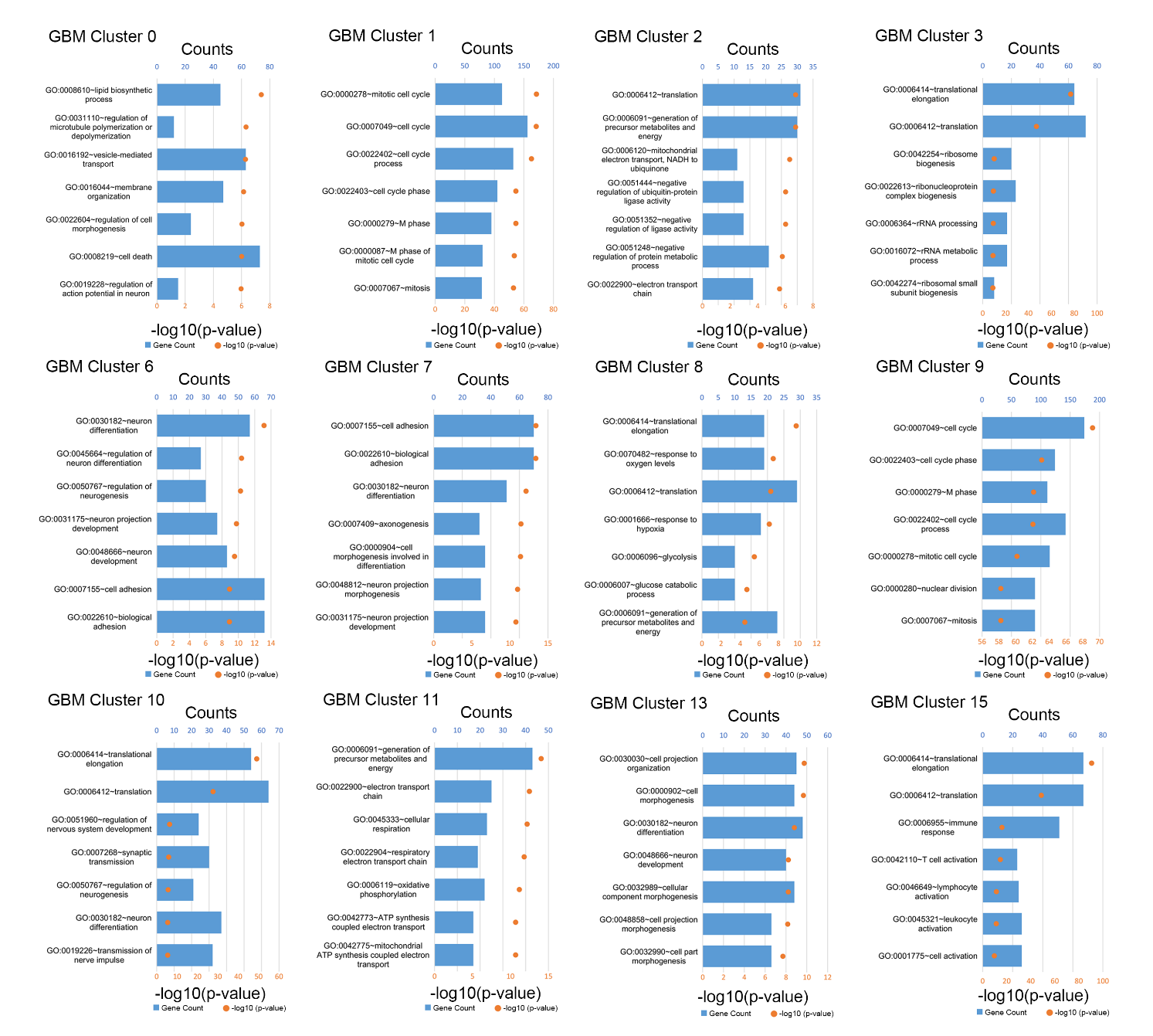


**Supplementary Figure 4.** Biological function enrichment analysis for tumor cells in the surgical specimens.


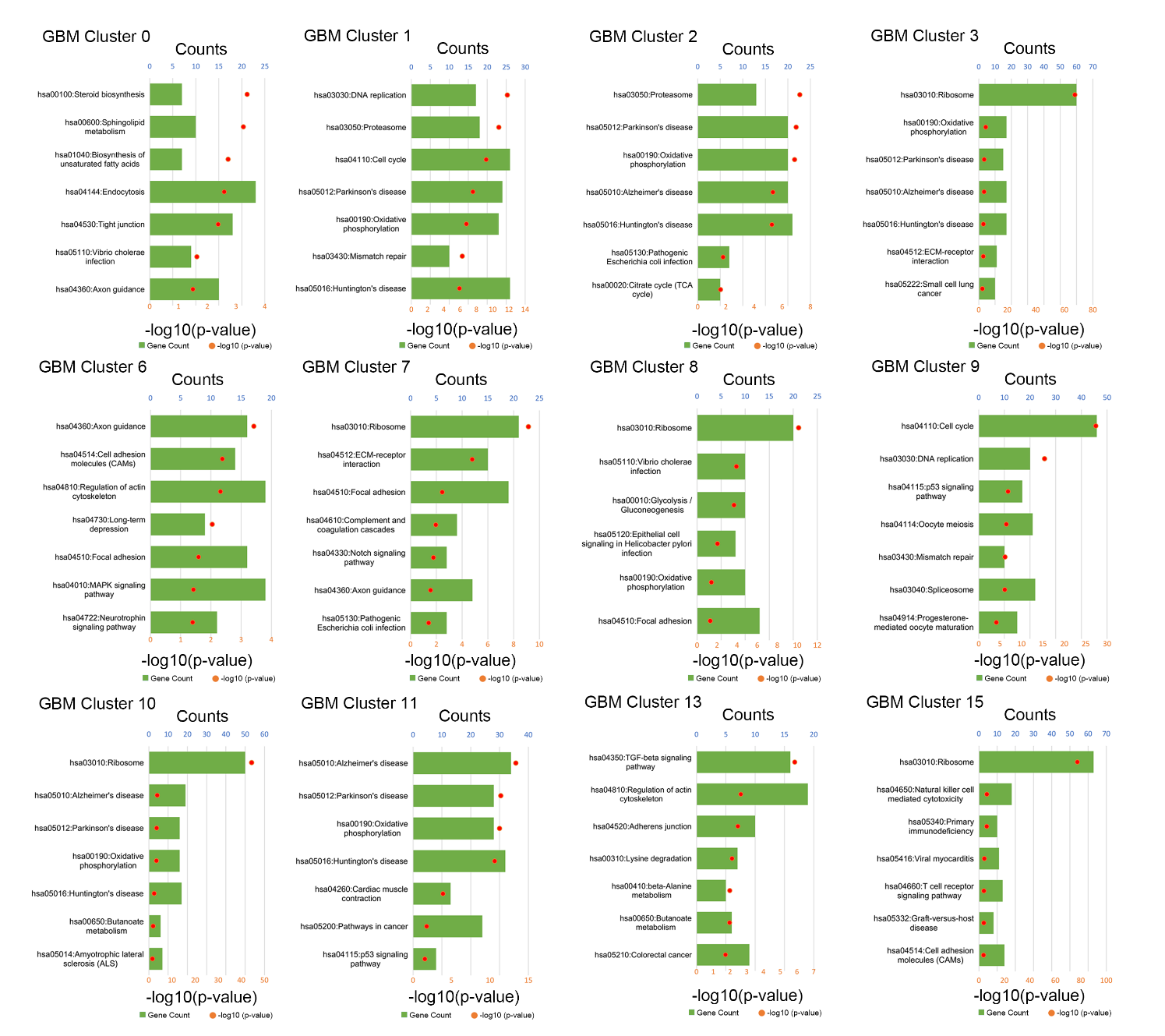


**Supplementary Figure 5.** Pathway enrichment analysis for tumor cells in the surgical specimens.


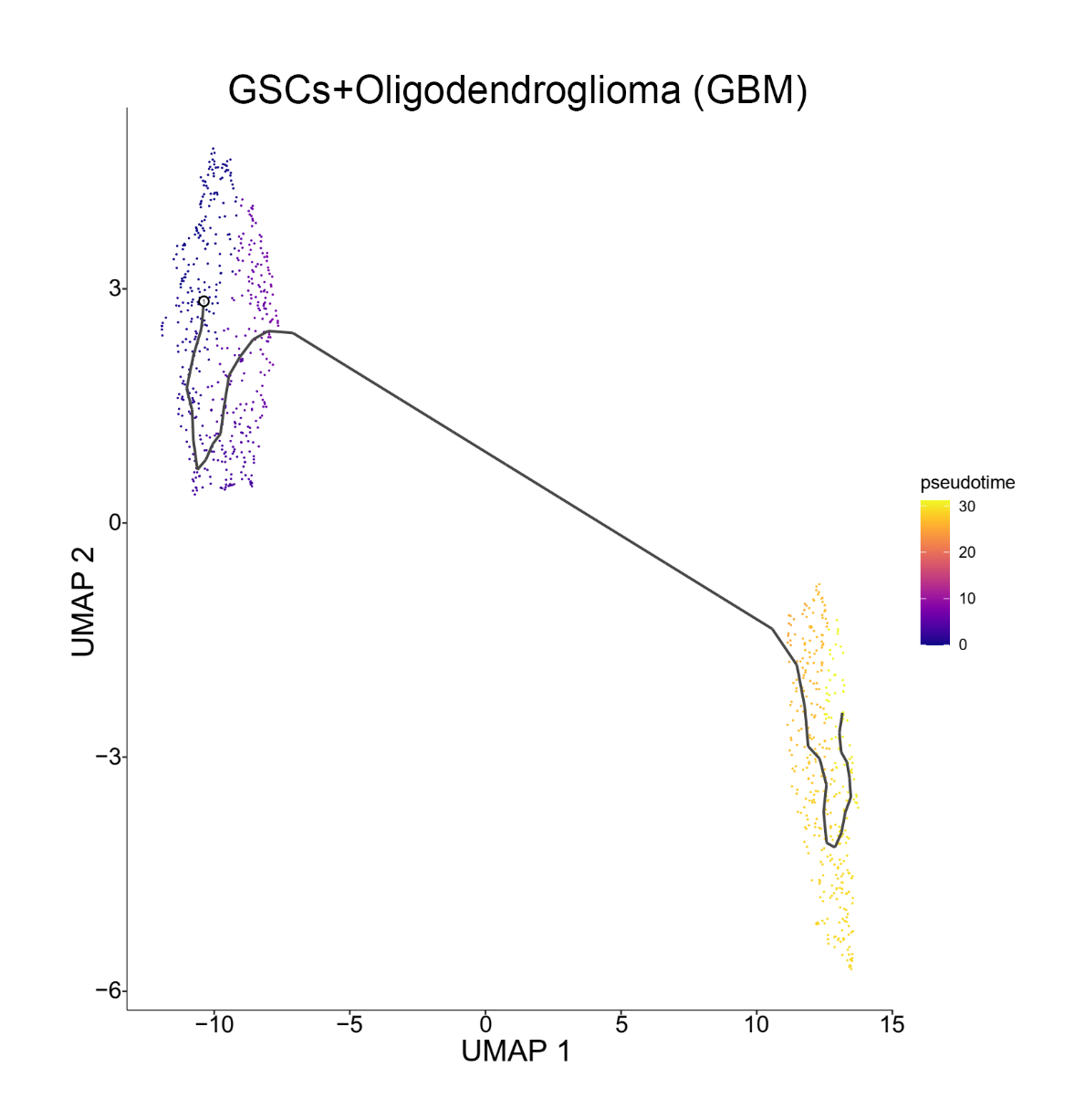


**Supplementary Figure 6.** The pseudo-time sequence of evolution of glioma stem cells and oligodendrocytes, in the surgical specimens.


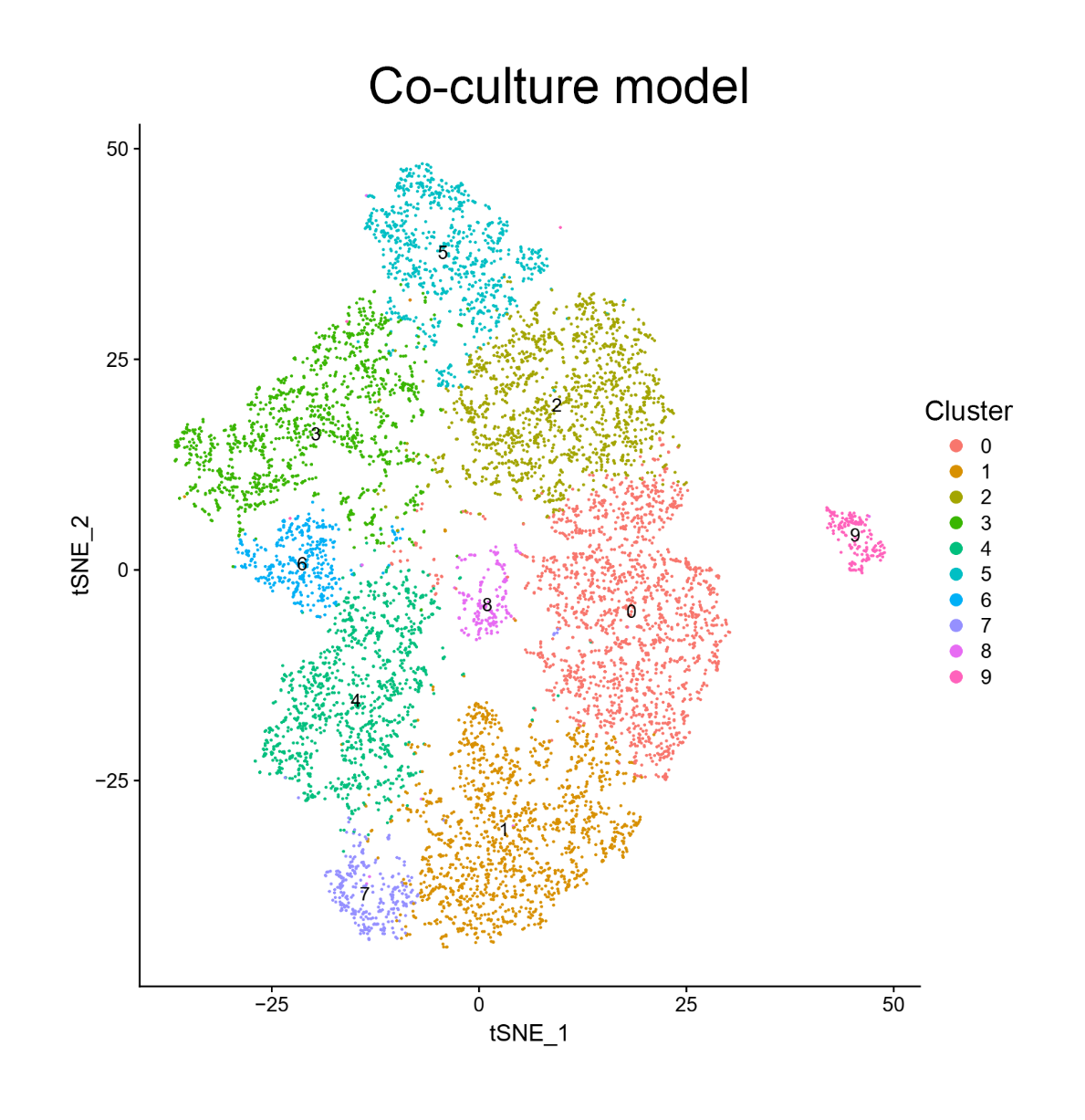


**Supplementary Figure 7.** The sub-groups of cells in the co-culture model by tSNE method.

## Supplementary Table S1

| **PC_ 1** | **Correlation** | **Cell Type** |  | **PC_ 2** | **Correlation** | **Cell Type** |
| --- | --- | --- | --- | --- | --- | --- |
| MAG | Positive | Oligodendrocyte |  | LGALS3 | Positive | NA |
| ERMN | Positive | Oligodendrocyte |  | S100A10 | Positive | NA |
| PPP1R14A | Positive | Oligodendrocyte |  | CHI3L1 | Positive | NA |
| TF | Positive | NA |  | EFEMP1 | Positive | Astrocyte |
| TMEM144 | Positive | NA |  | AEBP1 | Positive | NA |
| ENPP2 | Positive | NA |  | GFAP | Positive | Astrocyte |
| MBP | Positive | Oligodendrocyte |  | CLU | Positive | Astrocyte |
| CARNS1 | Positive | NA |  | IFITM3 | Positive | NA |
| MOBP | Positive | Oligodendrocyte |  | CD44 | Positive | Cancer cell |
| MAL | Positive | Oligodendrocyte |  | MT1X | Positive | Astrocyte |
| PTGDS | Positive | NA |  | GJA1 | Positive | NA |
| MOG | Positive | NA |  | AQP4 | Positive | Astrocyte |
| SPOCK3 | Positive | Neuron |  | NAMPT | Positive | NA |
| CNDP1 | Positive | NA |  | C1R | Positive | NA |
| CAPN3 | Positive | NA |  | IGFBP5 | Positive | Neuron |
| PLP1 | Positive | Oligodendrocyte |  | C1S | Positive | NA |
| KLK6 | Positive | Oligodendrocyte |  | EMP1 | Positive | NA |
| TMEM125 | Positive | NA |  | S100A6 | Positive | NA |
| RNASE1 | Positive | NA |  | SLC4A4 | Positive | Astrocyte |
| EDIL3 | Positive | NA |  | VEGFA | Positive | NA |
| HSPA2 | Positive | NA |  | COL6A1 | Positive | Neuron |
| EVI2A | Positive | NA |  | RDH10 | Positive | NA |
| APLP1 | Positive | NA |  | S100A11 | Positive | Macrophage |
| APOD | Positive | Oligodendrocyte |  | F3 | Positive | Astrocyte |
| PIP4K2A | Positive | NA |  | IGFBP7 | Positive | Astrocyte |
| SLC31A2 | Positive | NA |  | SERPINA3 | Positive | Astrocyte |
| TUBB4A | Positive | Oligodendrocyte |  | EPAS1 | Positive | Astrocyte |
| MYRF | Positive | NA |  | SERPING1 | Positive | NA |
| UGT8 | Positive | Oligodendrocyte |  | SOD2 | Positive | NA |
| CNP | Positive | Oligodendrocyte |  | ATP1B2 | Positive | Astrocyte |
| SEC61G | Negative | NA |  | TOP2A | Negative | NA |
| CST3 | Negative | Astrocyte |  | FAM64A | Negative | NA |
| FABP7 | Negative | Oligodendrocyte |  | ASPM | Negative | NA |
| TIMP1 | Negative | NA |  | NUSAP1 | Negative | NA |
| LDHA | Negative | NA |  | UBE2C | Negative | NA |
| IGFBP2 | Negative | NA |  | PRC1 | Negative | NA |
| CKS2 | Negative | NA |  | CKAP2L | Negative | NA |
| NES | Negative | NA |  | BIRC5 | Negative | NA |
| HSPB1 | Negative | NA |  | MKI67 | Negative | NA |
| CENPF | Negative | NA |  | AURKB | Negative | NA |
| PTTG1 | Negative | NA |  | TPX2 | Negative | NA |
| NUSAP1 | Negative | NA |  | PBK | Negative | NA |
| TOP2A | Negative | NA |  | HJURP | Negative | NA |
| MT2A | Negative | Astrocyte |  | KIF2C | Negative | NA |
| HMGB2 | Negative | Stem cell |  | NUF2 | Negative | NA |
| BIRC5 | Negative | NA |  | CENPF | Negative | NA |
| PBK | Negative | NA |  | TACC3 | Negative | NA |
| GNAS | Negative | NA |  | CENPE | Negative | NA |
| H2AFZ | Negative | NA |  | UBE2T | Negative | NA |
| FAM64A | Negative | NA |  | CCNB2 | Negative | NA |
| SMS | Negative | NA |  | MAD2L1 | Negative | NA |
| PRC1 | Negative | NA |  | PTTG1 | Negative | NA |
| SMC4 | Negative | NA |  | CCNB1 | Negative | NA |
| GAP43 | Negative | Oligodendrocyte |  | NDC80 | Negative | NA |
| ATP1B2 | Negative | Astrocyte |  | SGO2 | Negative | NA |
| EGFR | Negative | Cancer stem cell |  | PLK1 | Negative | NA |
| HES1 | Negative | NA |  | ECT2 | Negative | NA |
| LGALS1 | Negative | NA |  | CDCA8 | Negative | NA |
| HMGB3 | Negative | NA |  | CDCA3 | Negative | NA |
| UBE2T | Negative | NA |  | HMGB2 | Negative | Stem cell |

## Supplementary Table S2

Cell markers of clusters in GBM.

| **Gene Symbol** | **p_val_adj** | **cluster** | **Cell type** |
| --- | --- | --- | --- |
| SPOCK3 | 0 | 0 | Neuron |
| GRM3 | 0 | 0 | Oligodendrocyte |
| MAG | 7.50E-283 | 0 | Oligodendrocyte |
| ASPA | 2.33E-281 | 0 | Oligodendrocyte |
| GJB1 | 4.65E-274 | 0 | Oligodendrocyte |
| MOBP | 6.84E-272 | 0 | Oligodendrocyte |
| MAL | 3.53E-266 | 0 | Oligodendrocyte |
| FA2H | 1.15E-255 | 0 | Oligodendrocyte |
| ERMN | 2.07E-255 | 0 | Oligodendrocyte |
| LGI3 | 6.59E-255 | 0 | Oligodendrocyte |
| PPP1R14A | 7.53E-248 | 0 | Oligodendrocyte |
| KLK6 | 4.76E-243 | 0 | Oligodendrocyte |
| TUBB4A | 6.93E-241 | 0 | Oligodendrocyte |
| OPALIN | 7.33E-240 | 0 | Oligodendrocyte |
| UGT8 | 2.20E-238 | 0 | Oligodendrocyte |
| GPR37 | 7.88E-224 | 0 | Oligodendrocyte |
| CLDN11 | 6.93E-221 | 0 | Oligodendrocyte |
| MBP | 5.46E-220 | 0 | Oligodendrocyte |
| APOD | 6.17E-217 | 0 | Oligodendrocyte |
| CNP | 1.31E-214 | 0 | Oligodendrocyte |
| PLP1 | 7.74E-214 | 0 | Oligodendrocyte |
| CDK18 | 3.95E-213 | 0 | Oligodendrocyte |
| SEC14L5 | 7.12E-212 | 0 | Oligodendrocyte |
| CRYAB | 5.58E-211 | 0 | Astrocyte |
| PLEKHB1 | 1.78E-199 | 0 | Oligodendrocyte |
| ANLN | 5.52E-199 | 0 | Astrocyte |
| CLMN | 2.66E-188 | 0 | Neuron |
| OMG | 2.26E-185 | 0 | Oligodendrocyte |
| SIRT2 | 2.83E-184 | 0 | Oligodendrocyte |
| SCD | 1.41E-183 | 0 | Oligodendrocyte |
| GSN | 4.69E-183 | 0 | Oligodendrocyte |
| ZDHHC9 | 3.93E-181 | 0 | Oligodendrocyte |
| S100B | 2.47E-162 | 0 | Glial cell |
| KCNH8 | 1.43E-154 | 0 | Oligodendrocyte |
| FTH1 | 9.49E-153 | 0 | Astrocyte |
| MAP6D1 | 1.63E-150 | 0 | Oligodendrocyte |
| BIN1 | 3.16E-146 | 0 | Glial cell |
| SYNGR2 | 3.18E-143 | 0 | Macrophage |
| TMEFF2 | 5.25E-142 | 0 | Oligodendrocyte |
| ADAMTS4 | 8.31E-132 | 0 | Oligodendrocyte |
| LPAR1 | 9.58E-132 | 0 | Oligodendrocyte |
| CTNNA3 | 1.82E-130 | 0 | Neuron |
| RAB33A | 2.30E-125 | 0 | Oligodendrocyte |
| PHACTR3 | 1.43E-121 | 0 | Oligodendrocyte |
| KCNK1 | 7.16E-119 | 0 | Neuron |
| TSC22D4 | 2.73E-116 | 0 | Astrocyte |
| ANKRD18A | 6.48E-108 | 0 | Neuron |
| RTKN | 1.06E-107 | 0 | Oligodendrocyte |
| SPOCK1 | 2.22E-104 | 0 | Astrocyte |
| GLUL | 7.31E-95 | 0 | Astrocyte |
| MARCKSL1 | 6.80E-93 | 0 | Oligodendrocyte |
| PHLDB1 | 5.36E-91 | 0 | Oligodendrocyte |
| DHCR24 | 5.78E-91 | 0 | Oligodendrocyte |
| MAP1A | 2.29E-87 | 0 | Oligodendrocyte |
| NDRG2 | 3.95E-82 | 0 | Astrocyte |
| CYTH1 | 1.23E-77 | 0 | Macrophage |
| PAQR6 | 1.21E-76 | 0 | Astrocyte |
| PLD1 | 3.83E-68 | 0 | Neuron |
| TLE4 | 3.85E-66 | 0 | Neuron |
| AMOTL2 | 3.92E-64 | 0 | Oligodendrocyte |
| SERINC5 | 7.99E-60 | 0 | Oligodendrocyte |
| LGR5 | 9.96E-58 | 0 | Stem cell |
| EGLN3 | 2.84E-55 | 0 | Astrocyte |
| DPYSL5 | 5.87E-53 | 0 | Neuron |
| NFASC | 9.37E-49 | 0 | Neuron |
| PREX1 | 3.96E-45 | 0 | Neuron |
| ZCCHC24 | 1.06E-44 | 0 | Oligodendrocyte |
| OLIG1 | 3.94E-44 | 0 | Oligodendrocyte |
| SHISA4 | 2.74E-40 | 0 | Oligodendrocyte |
| SEMA6A | 8.96E-38 | 0 | Astrocyte |
| TOB2 | 1.01E-36 | 0 | Astrocyte |
| LMF1 | 2.28E-35 | 0 | Oligodendrocyte |
| DHCR7 | 2.16E-34 | 0 | Oligodendrocyte |
| TM7SF2 | 7.32E-32 | 0 | Oligodendrocyte |
| CXADR | 3.33E-30 | 0 | Oligodendrocyte |
| SNX22 | 2.08E-27 | 0 | Oligodendrocyte |
| SHC4 | 7.88E-27 | 0 | Oligodendrocyte |
| P2RX7 | 3.62E-26 | 0 | Oligodendrocyte |
| ZEB2 | 1.02E-24 | 0 | Oligodendrocyte |
| COBL | 9.67E-23 | 0 | Neuron |
| SNX1 | 6.64E-22 | 0 | Oligodendrocyte |
| PGRMC1 | 5.41E-19 | 0 | Oligodendrocyte |
| DNAJB1 | 1.29E-18 | 0 | Astrocyte |
| POLR2F | 1.51E-12 | 0 | Oligodendrocyte |
| TMEM123 | 5.65E-11 | 0 | Macrophage |
| HIP1 | 1.70E-06 | 0 | Oligodendrocyte |
| TNS1 | 4.86E-06 | 0 | Astrocyte |
| HEPACAM | 9.80E-05 | 0 | Astrocyte |
| FOSB | 0.000230641 | 0 | Astrocyte |
| SCAMP3 | 0.000315298 | 0 | Cancer stem cell |
| DLC1 | 0.00040011 | 0 | Neuron |
| JUNB | 0.001195546 | 0 | Astrocyte |
| MCAM | 0.045677106 | 0 | Stem cell |
| HMGB2 | 9.69E-164 | 1 | Stem cell |
| MKI67 | 6.73E-155 | 1 | Stem cell |
| GAS2L3 | 8.51E-103 | 1 | Neuron |
| FXYD7 | 1.15E-95 | 1 | Astrocyte |
| APOLD1 | 1.65E-90 | 1 | Endothelial cell |
| FABP7 | 3.94E-77 | 1 | Oligodendrocyte |
| HS3ST3B1 | 1.21E-71 | 1 | Neuron |
| PON2 | 1.54E-71 | 1 | Astrocyte |
| MEST | 5.63E-71 | 1 | Oligodendrocyte |
| EGFR | 3.19E-67 | 1 | Cancer stem cell |
| AGT | 2.82E-65 | 1 | Astrocyte |
| TUBB | 9.35E-65 | 1 | Oligodendrocyte |
| ANXA1 | 3.03E-59 | 1 | Neuron |
| FXYD5 | 1.55E-54 | 1 | Macrophage |
| MT2A | 1.01E-53 | 1 | Astrocyte |
| F3 | 4.07E-51 | 1 | Astrocyte |
| TMSB4X | 1.36E-46 | 1 | Oligodendrocyte |
| EDNRB | 3.25E-44 | 1 | Astrocyte |
| ASCL1 | 3.30E-44 | 1 | Cancer stem cell |
| EZR | 1.45E-43 | 1 | Astrocyte |
| EBP | 6.14E-40 | 1 | Oligodendrocyte |
| GRIK3 | 3.40E-39 | 1 | Neuron |
| NES | 4.13E-39 | 1 | Cancer stem cell |
| C1orf61 | 3.60E-36 | 1 | Astrocyte |
| NME1 | 4.43E-35 | 1 | Oligodendrocyte |
| HLA-A | 1.76E-32 | 1 | Neuron |
| VIM | 4.63E-27 | 1 | Stem cell |
| GAP43 | 6.34E-27 | 1 | Oligodendrocyte |
| CLU | 2.12E-26 | 1 | Astrocyte |
| LDHB | 3.26E-24 | 1 | Oligodendrocyte |
| ARL4A | 6.16E-22 | 1 | Oligodendrocyte |
| VOPP1 | 9.21E-21 | 1 | Macrophage |
| SEMA3E | 7.98E-113 | 2 | Neuron |
| SCG2 | 1.09E-73 | 2 | Astrocyte |
| TUBB3 | 2.02E-60 | 2 | Oligodendrocyte |
| GFAP | 5.12E-57 | 2 | Astrocyte |
| ITGB8 | 2.84E-54 | 2 | Neuron |
| ARSJ | 3.18E-49 | 2 | Neuron |
| JAG1 | 4.25E-44 | 2 | Neuron |
| RYR3 | 6.74E-44 | 2 | Neuron |
| COL6A1 | 2.26E-38 | 2 | Neuron |
| TMSB10 | 3.25E-38 | 2 | Oligodendrocyte |
| ITGA7 | 8.59E-38 | 2 | Astrocyte |
| ATP1B2 | 7.12E-31 | 2 | Astrocyte |
| COX7C | 4.25E-30 | 2 | Oligodendrocyte |
| PPP1R1C | 1.09E-29 | 2 | Neuron |
| BBOX1 | 1.23E-29 | 2 | Astrocyte |
| LHFPL3 | 1.76E-23 | 2 | Oligodendrocyte |
| PHLDA1 | 2.01E-22 | 2 | Oligodendrocyte |
| RGMA | 3.28E-22 | 2 | Astrocyte |
| SOX4 | 1.12E-21 | 2 | Cancer stem cell |
| SOX9 | 9.48E-21 | 2 | Astrocyte |
| GPR37L1 | 2.97E-15 | 2 | Oligodendrocyte |
| ANGPTL2 | 3.76E-14 | 2 | Oligodendrocyte |
| GRIA3 | 1.73E-13 | 2 | Neuron |
| DTNA | 1.13E-12 | 2 | Astrocyte |
| PDE4B | 2.39E-10 | 2 | Macrophage |
| PDGFD | 5.35E-78 | 3 | Neuron |
| SRPX | 1.84E-70 | 3 | Astrocyte |
| MLC1 | 3.95E-67 | 3 | Astrocyte |
| IGFBP5 | 2.72E-62 | 3 | Neuron |
| SLC4A4 | 6.28E-62 | 3 | Astrocyte |
| RPL13A | 2.41E-60 | 3 | Oligodendrocyte |
| LAMA2 | 1.43E-57 | 3 | Neuron |
| AQP4 | 6.97E-55 | 3 | Astrocyte |
| CST3 | 9.14E-55 | 3 | Astrocyte |
| SPRY1 | 2.93E-42 | 3 | Glial cell |
| POSTN | 1.99E-41 | 3 | Neuron |
| CCND2 | 5.82E-33 | 3 | Cancer stem cell |
| EPAS1 | 1.28E-32 | 3 | Astrocyte |
| SLC1A2 | 6.56E-31 | 3 | Astrocyte |
| LHFP | 6.58E-31 | 3 | Astrocyte |
| KIF21A | 2.37E-27 | 3 | Astrocyte |
| SLC6A11 | 1.36E-24 | 3 | Neuron |
| CDC42EP4 | 3.94E-24 | 3 | Astrocyte |
| ARHGEF26 | 5.13E-24 | 3 | Astrocyte |
| CNR1 | 1.82E-22 | 3 | Neuron |
| DCN | 4.29E-22 | 3 | Neuron |
| P2RY1 | 3.93E-20 | 3 | Astrocyte |
| CA12 | 2.59E-19 | 3 | Astrocyte |
| EFEMP1 | 3.93E-19 | 3 | Astrocyte |
| RPL31 | 1.28E-17 | 3 | Oligodendrocyte |
| PDLIM5 | 2.26E-17 | 3 | Neuron |
| HEY1 | 3.82E-16 | 3 | Astrocyte |
| RPS2 | 5.06E-15 | 3 | Oligodendrocyte |
| WSCD1 | 3.87E-14 | 3 | Oligodendrocyte |
| SOX2 | 7.11E-14 | 3 | Cancer stem cell |
| RRBP1 | 8.83E-14 | 3 | Neuron |
| EPN2 | 1.55E-11 | 3 | Oligodendrocyte |
| SLC1A3 | 1.64E-11 | 3 | Astrocyte |
| ATP13A4 | 2.26E-11 | 3 | Astrocyte |
| EEF2 | 3.57E-10 | 3 | Oligodendrocyte |
| RPS23 | 3.53E-09 | 3 | Oligodendrocyte |
| LPL | 3.13E-08 | 3 | Astrocyte |
| CHST8 | 2.51E-05 | 3 | Neuron |
| COL5A2 | 7.11E-05 | 3 | Neuron |
| SPARCL1 | 1 | 3 | Astrocyte |
| IGFBP7 | 1 | 3 | Astrocyte |
| P2RY12 | 2.13E-183 | 4 | Glial cell |
| SIGLEC8 | 3.76E-159 | 4 | Glial cell |
| CSF1R | 1.81E-143 | 4 | Glial cell |
| ADORA3 | 1.26E-135 | 4 | Glial cell |
| A2M | 9.12E-135 | 4 | Endothelial cell |
| AIF1 | 2.62E-122 | 4 | Macrophage |
| CD68 | 1.75E-120 | 4 | Glial cell |
| LAPTM5 | 2.43E-113 | 4 | Glial cell |
| SORL1 | 2.30E-112 | 4 | Astrocyte |
| BHLHE41 | 7.68E-112 | 4 | Glial cell |
| CD83 | 1.25E-111 | 4 | Glial cell |
| TAL1 | 2.43E-107 | 4 | Glial cell |
| PTPRC | 3.12E-105 | 4 | Stem cell |
| CD74 | 1.36E-104 | 4 | Macrophage |
| HLA-DMB | 1.40E-102 | 4 | Macrophage |
| ZFP36 | 5.83E-101 | 4 | Astrocyte |
| FOS | 2.98E-95 | 4 | Astrocyte |
| MS4A7 | 1.78E-93 | 4 | Macrophage |
| CYBB | 2.51E-91 | 4 | Macrophage |
| KLF2 | 6.82E-91 | 4 | Glial cell |
| CD86 | 1.52E-88 | 4 | Astrocyte |
| SGK1 | 6.23E-87 | 4 | Oligodendrocyte |
| HLA-DRA | 7.51E-86 | 4 | Glial cell |
| SFMBT2 | 6.83E-83 | 4 | Neuron |
| CD4 | 2.92E-77 | 4 | T cell |
| HLA-E | 4.07E-77 | 4 | Astrocyte |
| BTG2 | 1.69E-75 | 4 | Astrocyte |
| ADRB2 | 4.34E-74 | 4 | Glial cell |
| ZFP36L1 | 8.64E-74 | 4 | Astrocyte |
| NR4A2 | 4.15E-72 | 4 | Neuron |
| RCSD1 | 1.18E-70 | 4 | Neuron |
| IER2 | 2.83E-70 | 4 | Astrocyte |
| PSAP | 1.23E-66 | 4 | Astrocyte |
| CPVL | 3.44E-64 | 4 | Astrocyte |
| NAV3 | 4.93E-64 | 4 | Glial cell |
| CD14 | 7.01E-61 | 4 | Glial cell |
| ZFP36L2 | 2.37E-58 | 4 | Astrocyte |
| GPR183 | 2.22E-57 | 4 | Glial cell |
| ZFHX3 | 1.22E-56 | 4 | Neuron |
| RASGEF1C | 3.74E-53 | 4 | Neuron |
| NFKBIA | 3.71E-52 | 4 | Astrocyte |
| APOE | 2.10E-43 | 4 | Astrocyte |
| C5AR1 | 2.44E-42 | 4 | Astrocyte |
| RIN2 | 7.08E-42 | 4 | Neuron |
| RHOB | 2.26E-41 | 4 | Astrocyte |
| CD81 | 1.28E-38 | 4 | Stem cell |
| HLA-DRB5 | 2.14E-36 | 4 | Macrophage |
| SPINT2 | 3.10E-35 | 4 | Macrophage |
| MAFB | 2.20E-34 | 4 | Astrocyte |
| METTL7A | 2.96E-32 | 4 | Astrocyte |
| PECAM1 | 2.10E-31 | 4 | Endothelial cell |
| CXCR4 | 2.58E-26 | 4 | Stem cell |
| TSPO | 7.36E-26 | 4 | Macrophage |
| HMOX1 | 2.60E-24 | 4 | Cancer stem cell |
| JUN | 4.25E-21 | 4 | Astrocyte |
| MICAL1 | 1.63E-20 | 4 | Oligodendrocyte |
| LIMS1 | 2.27E-20 | 4 | Neuron |
| MS4A6A | 5.13E-18 | 4 | Macrophage |
| C10orf11 | 5.30E-17 | 4 | Neuron |
| PFKFB3 | 8.13E-17 | 4 | Astrocyte |
| RASSF4 | 6.88E-15 | 4 | Astrocyte |
| JUND | 1.07E-13 | 4 | Astrocyte |
| DNASE2 | 8.97E-13 | 4 | Astrocyte |
| TREM1 | 3.46E-12 | 4 | Macrophage |
| SAMHD1 | 2.81E-11 | 4 | Macrophage |
| PER1 | 1.82E-10 | 4 | Astrocyte |
| SWAP70 | 9.98E-08 | 4 | Neuron |
| BCL2A1 | 5.80E-07 | 4 | Glial cell |
| SYT6 | 9.24E-07 | 4 | Neuron |
| SH3BGRL | 3.75E-05 | 4 | Macrophage |
| TPCN1 | 0.000147747 | 4 | Astrocyte |
| NAP1L1 | 0.001459878 | 4 | Oligodendrocyte |
| ZFAND5 | 0.038572711 | 4 | Astrocyte |
| ATF3 | 1 | 4 | Astrocyte |
| ZNF703 | 1 | 4 | Neuron |
| MTSS1 | 1 | 4 | Oligodendrocyte |
| SALL1 | 1 | 4 | Glial cell |
| LYZ | 6.87E-129 | 5 | Macrophage |
| TGFBI | 5.94E-109 | 5 | Macrophage |
| CD93 | 4.17E-85 | 5 | Macrophage |
| CD163 | 2.21E-83 | 5 | Macrophage |
| GPR65 | 2.40E-66 | 5 | Macrophage |
| FGR | 8.14E-63 | 5 | Macrophage |
| METRNL | 2.78E-57 | 5 | Macrophage |
| ITGA4 | 2.92E-55 | 5 | Macrophage |
| KYNU | 1.38E-54 | 5 | Macrophage |
| EMB | 1.95E-54 | 5 | Macrophage |
| IL1RN | 5.91E-54 | 5 | Macrophage |
| DOK3 | 1.73E-52 | 5 | Macrophage |
| IFITM2 | 4.93E-49 | 5 | Macrophage |
| THBD | 3.01E-48 | 5 | Macrophage |
| FPR3 | 2.30E-42 | 5 | Macrophage |
| DPYD | 1.14E-41 | 5 | Oligodendrocyte |
| PLBD1 | 1.18E-37 | 5 | Macrophage |
| SLC2A3 | 1.94E-37 | 5 | Cancer stem cell |
| DSE | 8.01E-36 | 5 | Macrophage |
| RASSF3 | 4.85E-31 | 5 | Neuron |
| CD44 | 6.35E-31 | 5 | Cancer stem cell |
| HLA-DQA1 | 2.14E-30 | 5 | Macrophage |
| IQGAP1 | 5.57E-25 | 5 | Macrophage |
| OLFML2B | 1.37E-22 | 5 | Neuron |
| NRP1 | 2.07E-22 | 5 | Astrocyte |
| MXD1 | 3.00E-19 | 5 | Macrophage |
| CCL2 | 3.95E-18 | 5 | Glial cell |
| ABCA1 | 9.40E-11 | 5 | Astrocyte |
| NFIL3 | 8.16E-09 | 5 | Macrophage |
| CREM | 3.37E-07 | 5 | Macrophage |
| BHLHE40 | 4.14E-06 | 5 | Macrophage |
| PIM1 | 2.34E-05 | 5 | Macrophage |
| GADD45B | 0.000150474 | 5 | Astrocyte |
| HIF1A | 0.054689873 | 5 | Astrocyte |
| CIB1 | 0.247496545 | 5 | Macrophage |
| HPCAL1 | 0.249283255 | 5 | Neuron |
| EIF3L | 0.789528306 | 5 | Oligodendrocyte |
| HNMT | 1 | 5 | Astrocyte |
| PDGFRA | 2.52E-81 | 6 | Neuron |
| MAP2 | 1.93E-64 | 6 | Neuron |
| GABRB1 | 3.23E-60 | 6 | Astrocyte |
| LUZP2 | 1.16E-59 | 6 | Neuron |
| ATCAY | 2.82E-58 | 6 | Oligodendrocyte |
| KLRC2 | 1.42E-57 | 6 | Oligodendrocyte |
| GRIA2 | 8.39E-54 | 6 | Oligodendrocyte |
| SOX11 | 9.90E-51 | 6 | Cancer stem cell |
| PCDH15 | 1.31E-47 | 6 | Progenitor cell |
| MYT1 | 5.62E-43 | 6 | Oligodendrocyte |
| ASIC1 | 4.33E-39 | 6 | Oligodendrocyte |
| OPCML | 3.08E-38 | 6 | Oligodendrocyte |
| SERPINE2 | 2.87E-37 | 6 | Neuron |
| CPE | 5.04E-37 | 6 | Astrocyte |
| NFIB | 6.86E-37 | 6 | Cancer stem cell |
| MMD2 | 3.33E-36 | 6 | Astrocyte |
| SCG3 | 4.87E-36 | 6 | Astrocyte |
| DLL3 | 9.20E-35 | 6 | Oligodendrocyte |
| LNX1 | 1.25E-33 | 6 | Neuron |
| LRRN1 | 6.47E-31 | 6 | Oligodendrocyte |
| FXYD6 | 1.13E-30 | 6 | Oligodendrocyte |
| NTRK2 | 1.69E-30 | 6 | Astrocyte |
| ADCYAP1R1 | 1.50E-27 | 6 | Astrocyte |
| THY1 | 7.82E-27 | 6 | Cancer stem cell |
| TCF4 | 5.35E-26 | 6 | Cancer stem cell |
| DCX | 8.11E-25 | 6 | Progenitor cell |
| IL33 | 1.56E-23 | 6 | Astrocyte |
| TSPAN18 | 3.53E-22 | 6 | Neuron |
| CDO1 | 1.96E-20 | 6 | Neuron |
| SEMA5A | 3.67E-20 | 6 | Neuron |
| OLIG2 | 6.09E-18 | 6 | Oligodendrocyte |
| NPPA | 7.85E-16 | 6 | Oligodendrocyte |
| PEA15 | 8.95E-16 | 6 | Astrocyte |
| TM4SF1 | 7.34E-15 | 6 | Endothelial cell |
| GRIK2 | 1.03E-14 | 6 | Oligodendrocyte |
| TAGLN3 | 1.10E-14 | 6 | Oligodendrocyte |
| GFRA1 | 1.30E-14 | 6 | Neuron |
| CXXC4 | 3.31E-14 | 6 | Neuron |
| TNK2 | 9.01E-14 | 6 | Oligodendrocyte |
| NXPH1 | 1.01E-13 | 6 | Neuron |
| GNG4 | 1.28E-13 | 6 | Neuron |
| TRIL | 2.32E-13 | 6 | Astrocyte |
| SOX8 | 1.48E-11 | 6 | Oligodendrocyte |
| LIMA1 | 1.07E-09 | 6 | Oligodendrocyte |
| CHD7 | 3.37E-08 | 6 | Cancer stem cell |
| NEUROD1 | 4.15E-07 | 6 | Progenitor cell |
| TCF12 | 3.67E-06 | 6 | Oligodendrocyte |
| CSPG4 | 3.88E-06 | 6 | Oligodendrocyte |
| PROM1 | 4.77E-06 | 6 | Cancer stem cell |
| MYO10 | 5.48E-05 | 6 | Neuron |
| SHD | 0.000370455 | 6 | Oligodendrocyte |
| RFX4 | 0.005351741 | 6 | Astrocyte |
| RAB2A | 0.006637156 | 6 | Oligodendrocyte |
| GRIA1 | 0.007890938 | 6 | Astrocyte |
| CREB5 | 0.008115239 | 6 | Neuron |
| DKK3 | 0.011582219 | 6 | Astrocyte |
| ALDOC | 0.012616515 | 6 | Astrocyte |
| RGMB | 0.024625452 | 6 | Oligodendrocyte |
| MAML2 | 0.034500383 | 6 | Oligodendrocyte |
| DLL1 | 0.03994456 | 6 | Oligodendrocyte |
| LIX1 | 0.04102892 | 6 | Astrocyte |
| NFIA | 0.049768819 | 6 | Astrocyte |
| GRIA4 | 0.052367427 | 6 | Oligodendrocyte |
| CD24 | 0.106117541 | 6 | Cancer stem cell |
| RGS12 | 0.155824728 | 6 | Neuron |
| SPON1 | 0.322007967 | 6 | Astrocyte |
| HIPK2 | 1 | 6 | Oligodendrocyte |
| TMEM255A | 1 | 6 | Neuron |
| RND3 | 1 | 6 | Astrocyte |
| PDE9A | 1 | 6 | Neuron |
| TIMP3 | 1 | 6 | Astrocyte |
| SULF1 | 2.81E-59 | 7 | Neuron |
| AFAP1L2 | 1.84E-57 | 7 | Oligodendrocyte |
| MEGF11 | 2.59E-55 | 7 | Progenitor cell |
| L1CAM | 1.77E-47 | 7 | Astrocyte |
| TOX | 2.65E-29 | 7 | Neuron |
| CHRDL1 | 1.01E-28 | 7 | Astrocyte |
| CTNNB1 | 5.53E-24 | 7 | Cancer stem cell |
| PRRX1 | 7.40E-18 | 7 | Neuron |
| SERPINA3 | 8.64E-17 | 7 | Astrocyte |
| NACA | 1.53E-15 | 7 | Oligodendrocyte |
| ACTG1 | 4.12E-15 | 7 | Oligodendrocyte |
| TSHZ2 | 1.13E-12 | 7 | Neuron |
| NOTCH1 | 2.67E-10 | 7 | Stem cell |
| IFITM10 | 1.12E-09 | 7 | Neuron |
| GPR17 | 1.46E-05 | 7 | Oligodendrocyte |
| CDH13 | 0.002512896 | 7 | Oligodendrocyte |
| LRRTM3 | 0.05873943 | 7 | Neuron |
| COL20A1 | 1 | 7 | Neuron |
| MT1X | 1.21E-43 | 8 | Astrocyte |
| ENO2 | 3.00E-36 | 8 | Neuron |
| SLC16A1 | 2.18E-19 | 8 | Cancer stem cell |
| PCSK5 | 1.07E-15 | 8 | Neuron |
| DOK5 | 4.78E-15 | 8 | Astrocyte |
| PCDH8 | 7.74E-12 | 8 | Neuron |
| NMU | 1.23E-41 | 9 | Neuron |
| CCDC141 | 1.05E-20 | 9 | Neuron |
| TMEM97 | 5.62E-13 | 9 | Oligodendrocyte |
| VIPR2 | 2.31E-86 | 10 | Neuron |
| RGR | 1.63E-76 | 10 | Oligodendrocyte |
| FERMT1 | 7.41E-74 | 10 | Oligodendrocyte |
| KCNIP2 | 8.07E-71 | 10 | Astrocyte |
| KCNIP3 | 4.88E-69 | 10 | Oligodendrocyte |
| ST6GAL2 | 6.03E-59 | 10 | Astrocyte |
| PID1 | 2.50E-45 | 10 | Oligodendrocyte |
| CRTAC1 | 1.73E-32 | 10 | Neuron |
| NEU4 | 1.11E-30 | 10 | Oligodendrocyte |
| ID1 | 1.01E-25 | 10 | Astrocyte |
| ACSL6 | 8.55E-25 | 10 | Astrocyte |
| MTHFD2 | 6.88E-23 | 10 | Astrocyte |
| FAU | 1.19E-14 | 10 | Oligodendrocyte |
| TBC1D10A | 8.10E-11 | 10 | Astrocyte |
| LRIG1 | 2.05E-10 | 10 | Astrocyte |
| DGKG | 5.67E-07 | 10 | Astrocyte |
| UQCRB | 5.61E-05 | 10 | Oligodendrocyte |
| CAMK2G | 5.73E-05 | 10 | Neuron |
| GATSL3 | 0.001407443 | 10 | Astrocyte |
| CRB1 | 0.010659666 | 10 | Oligodendrocyte |
| EIF3E | 0.057223918 | 10 | Oligodendrocyte |
| ID4 | 0.46218349 | 10 | Astrocyte |
| EEF1B2 | 1 | 10 | Oligodendrocyte |
| TSPAN12 | 2.01E-45 | 11 | Astrocyte |
| CPNE5 | 2.43E-40 | 11 | Astrocyte |
| CRIM1 | 1.08E-22 | 11 | Neuron |
| ITGB4 | 1.21E-15 | 11 | Astrocyte |
| LYPD1 | 8.91E-14 | 11 | Astrocyte |
| CADM1 | 1.31E-12 | 11 | Cancer stem cell |
| BMPR1B | 1.55E-09 | 11 | Astrocyte |
| NTNG1 | 1.76E-08 | 11 | Neuron |
| SLC7A11 | 6.12E-08 | 11 | Neuron |
| LRRC8A | 0.00459197 | 11 | Astrocyte |
| ITGA6 | 0.007613761 | 11 | Stem cell |
| HPSE2 | 6.39E-24 | 12 | Neuron |
| CD40 | 1.02E-17 | 12 | Astrocyte |
| ISG20 | 1.49E-05 | 12 | T cell |
| FOLR1 | 2.12E-177 | 13 | Neuron |
| ALDH1A1 | 5.19E-25 | 13 | Cancer stem cell |
| PLTP | 2.69E-24 | 13 | Astrocyte |
| GLIS3 | 6.75E-20 | 13 | Astrocyte |
| EXPH5 | 4.03E-16 | 13 | Neuron |
| HSPB8 | 2.29E-13 | 13 | Astrocyte |
| CHST9 | 2.27E-09 | 13 | Astrocyte |
| LTBP1 | 0.00182639 | 13 | Neuron |
| GRAMD3 | 0.003068589 | 13 | Astrocyte |
| PAX6 | 0.003252548 | 13 | Stem cell |
| NHSL1 | 1 | 13 | Astrocyte |
| CYBRD1 | 1 | 13 | Astrocyte |
| DLX5 | 6.60E-89 | 14 | Neuron |
| KIT | 1.43E-84 | 14 | Neuron |
| DLX6 | 1.55E-71 | 14 | Neuron |
| ADAMTSL1 | 8.02E-55 | 14 | Neuron |
| DLX1 | 1.48E-53 | 14 | Neuron |
| GLI3 | 6.87E-15 | 14 | Astrocyte |
| DLX2 | 1.52E-14 | 14 | Neuron |
| C2orf27A | 0.000146405 | 14 | Oligodendrocyte |
| RPSAP58 | 0.00089585 | 14 | Oligodendrocyte |
| NPM1 | 0.010444735 | 14 | Oligodendrocyte |
| CD3E | 1.16E-174 | 15 | T cell |
| CXCR3 | 1.48E-109 | 15 | T cell |
| CD3G | 2.09E-94 | 15 | T cell |
| CD3D | 2.70E-75 | 15 | T cell |
| PTPN7 | 9.16E-12 | 15 | Macrophage |
| PLAC8 | 0.662293628 | 15 | Macrophage |

Cell markers of clusters in GSCs.

| **Gene Symbol** | **p_val_adj** | **cluster** | **Cell type** |
| --- | --- | --- | --- |
| IFITM2 | 3.16E-194 | 0 | Macrophage |
| NME1 | 2.08E-165 | 0 | Oligodendrocyte |
| EEF1B2 | 1.07E-144 | 0 | Oligodendrocyte |
| AIF1 | 1.18E-94 | 0 | Macrophage |
| PLAC8 | 3.08E-86 | 0 | Macrophage |
| TUBB | 0 | 1 | Oligodendrocyte |
| MKI67 | 0 | 1 | Stem cell |
| HMGB2 | 0 | 1 | Stem cell |
| ACAT2 | 2.23E-186 | 1 | Oligodendrocyte |
| TESC | 4.52E-153 | 1 | Neuron |
| FDPS | 3.62E-110 | 1 | Oligodendrocyte |
| MT2A | 3.07E-81 | 1 | Astrocyte |
| FTH1 | 3.22E-69 | 1 | Astrocyte |
| NACA | 2.82E-261 | 2 | Oligodendrocyte |
| GNB2L1 | 8.76E-235 | 2 | Oligodendrocyte |
| HLA-E | 3.69E-137 | 2 | Astrocyte |
| MAL | 4.46E-123 | 2 | Oligodendrocyte |
| EIF3E | 2.25E-112 | 2 | Oligodendrocyte |
| SPINT2 | 1.27E-71 | 2 | Macrophage |
| TXK | 5.46E-55 | 2 | Neuron |
| CD4 | 0 | 3 | T cell |
| KLF2 | 1.43E-207 | 3 | Glial cell |
| LIMS1 | 5.99E-190 | 3 | Neuron |
| JUNB | 1.94E-126 | 3 | Astrocyte |
| HLA-A | 2.66E-114 | 3 | Neuron |
| ISG20 | 5.64E-101 | 3 | T cell |
| FXYD5 | 2.47E-90 | 3 | Macrophage |
| ITGB1 | 5.54E-89 | 3 | Stem cell |
| GPR183 | 5.15E-84 | 3 | Glial cell |
| LAPTM5 | 1.17E-69 | 3 | Glial cell |
| BTG2 | 1.60E-67 | 3 | Astrocyte |
| SLC2A3 | 8.46E-67 | 3 | Cancer stem cell |
| CYTH1 | 2.65E-61 | 3 | Macrophage |
| CXCR4 | 3.13E-59 | 3 | Stem cell |
| CD44 | 1.07E-48 | 3 | Cancer stem cell |
| ZFP36 | 1.13E-45 | 3 | Astrocyte |
| ITGA4 | 3.96E-41 | 3 | Macrophage |
| ANXA1 | 2.49E-28 | 3 | Neuron |
| ZFP36L2 | 2.41E-25 | 3 | Astrocyte |
| IER2 | 3.05E-16 | 3 | Astrocyte |
| PDE4B | 9.94E-16 | 3 | Macrophage |
| NFKBIA | 6.35E-06 | 3 | Astrocyte |
| HLA-DRA | 0.71420706 | 3 | Glial cell |
| HLA-DRB5 | 0 | 4 | Macrophage |
| CD74 | 0 | 4 | Macrophage |
| HLA-DQA1 | 0 | 4 | Macrophage |
| CCL4 | 1.37E-289 | 4 | Glial cell |
| PHLDA1 | 1.03E-283 | 4 | Oligodendrocyte |
| EBP | 1.21E-209 | 4 | Oligodendrocyte |
| HLA-DMB | 2.59E-182 | 4 | Macrophage |
| ARG2 | 1.77E-163 | 4 | Macrophage |
| VIM | 8.84E-143 | 4 | Stem cell |
| LIMA1 | 1.73E-100 | 4 | Oligodendrocyte |
| CD81 | 3.15E-97 | 4 | Stem cell |
| IQGAP1 | 2.61E-82 | 4 | Macrophage |
| PTPN7 | 9.70E-57 | 4 | Macrophage |
| FAU | 5.78E-158 | 5 | Oligodendrocyte |
| RPL13A | 4.53E-109 | 5 | Oligodendrocyte |
| RPL31 | 2.52E-74 | 5 | Oligodendrocyte |
| SORL1 | 5.14E-05 | 5 | Astrocyte |
| PCED1B | 0.000467874 | 5 | Neuron |
| BCL11B | 0.002817894 | 5 | Neuron |
| METRNL | 1.33E-33 | 6 | Macrophage |
| PTPRC | 1.01E-32 | 6 | Stem cell |
| CD3D | 3.71E-20 | 6 | T cell |
| BHLHE40 | 4.17E-12 | 6 | Macrophage |
| EMB | 1.13E-05 | 6 | Macrophage |
| SFMBT2 | 0.000131249 | 6 | Neuron |
| TSC22D4 | 0.000550441 | 6 | Astrocyte |
| JUN | 0.400054288 | 6 | Astrocyte |
| ZFP36L1 | 1 | 6 | Astrocyte |
| THEMIS | 1 | 6 | Neuron |
| SIRT2 | 1 | 6 | Oligodendrocyte |
| ANLN | 1.31E-141 | 7 | Astrocyte |
| ACTG1 | 3.54E-125 | 7 | Oligodendrocyte |
| CREM | 1.29E-26 | 7 | Macrophage |
| TMEM123 | 3.48E-05 | 8 | Macrophage |
| TMEM97 | 1 | 8 | Oligodendrocyte |
| TMSB4X | 9.22E-27 | 9 | Oligodendrocyte |
| TUBB3 | 1.67E-12 | 9 | Oligodendrocyte |
| SH3BGRL | 4.61E-10 | 9 | Macrophage |
| POLR2F | 1.73E-06 | 9 | Oligodendrocyte |
| MT1X | 0.050548138 | 9 | Astrocyte |
| TSPO | 0.159238158 | 9 | Macrophage |
| NPM1 | 1 | 9 | Oligodendrocyte |
